# Alpha-synuclein shapes monocyte and macrophage cell biology and functions by bridging alterations of autophagy and inflammatory pathways

**DOI:** 10.3389/fcell.2024.1421360

**Published:** 2024-07-05

**Authors:** Fiona Limanaqi, Silvia Zecchini, Pasquale Ogno, Valentina Artusa, Claudio Fenizia, Irma Saulle, Claudia Vanetti, Micaela Garziano, Sergio Strizzi, Daria Trabattoni, Mario Clerici, Mara Biasin

**Affiliations:** ^1^ Department of Biomedical and Clinical Sciences, University of Milan, Milan, Italy; ^2^ Department of Pathophysiology and Transplantation, University of Milan, Milan, Italy; ^3^ IRCCS Fondazione Don Carlo Gnocchi, Milan, Italy

**Keywords:** cell survival, cell toxicity, p62, lysosomes, cell migration, chemotaxis, phagocytosis, lipid droplets

## Abstract

**Introduction:** Abnormal spreading of alpha-synuclein (αS), a hallmark of Parkinson’s disease, is known to promote peripheral inflammation, which occurs in part via functional alterations in monocytes/macrophages. However, underlying intracellular mechanisms remain unclear.

**Methods:** Herein we investigate the subcellular, molecular, and functional effects of excess αS in human THP-1 monocytic cell line, THP-1-derived macrophages, and at least preliminarily, in primary monocyte-derived macrophages (MDMs). In cells cultured w/wo recombinant αS (1 μM) for 4 h and 24 h, by Confocal microscopy, Western Blot, RT-qPCR, Elisa, and Flow Cytometry we assessed: i) αS internalization; ii) cytokine/chemokine expression/secretion, and C–C motif chemokine receptor 2 (CCR2) levels; iii) autophagy (LC3II/I, LAMP1/LysoTracker, p62, pS6/total S6); and iv) lipid droplets (LDs) accumulation, and cholesterol pathway gene expression. Transwell migration assay was employed to measure THP-1 cell migration/chemotaxis, while FITC-IgG-bead assay was used to analyze phagocytic capacity, and the fate of phagocytosed cargo in THP-1-derived macrophages.

**Results:** Extracellular αS was internalized by THP-1 cells, THP-1-derived macrophages, and MDMs. In THP1 cells, αS induced a general pro-inflammatory profile and conditioned media from αS-exposed THP-1 cells potently attracted unstimulated cells. However, CCL2 secretion peaked at 4 h αS, consistent with early internalization of its receptor CCR2, while this was blunted at 24 h αS exposure, when CCR2 recycled back to the plasma membrane. Again, 4 h αS-exposed THP-1 cells showed increased spontaneous migration, while 24 h αS-exposed cells showed reduced chemotaxis. This occurred in the absence of cell toxicity and was associated with upregulation of autophagy/lysosomal markers, suggesting a pro-survival/tolerance mechanism against stress-related inflammation. Instead, in THP-1-derived macrophages, αS time-dependently potentiated the intracellular accumulation, and release of pro-inflammatory mediators. This was accompanied by mild toxicity, reduced autophagy-lysosomal markers, defective LDs formation, as well as impaired phagocytosis, and the appearance of stagnant lysosomes engulfed with phagocytosed cargo, suggesting a status of macrophage exhaustion reminiscent of hypophagia.

**Discussion:** In summary, despite an apparently similar pro-inflammatory phenotype, monocytes and macrophages respond differently to intracellular αS accumulation in terms of cell survival, metabolism, and functions. Our results suggest that in periphery, αS exerts cell- and context-specific biological effects bridging alterations of autophagy, lipid dynamics, and inflammatory pathways.

## 1 Introduction

Alpha-synuclein (αS), an intrinsically disordered, and highly dynamic protein which is prone to misfold and aggregate in nerve cells, is now widely recognized as a hallmark of neurodegenerative synucleinopathies including Parkinson’s disease (PD) and multiple system atrophy (MSA). While exhaustive evidence has been provided on the pathophysiological roles of αS within nerve and glial cells (Burré et al., 2018; Ryskalin et al., 2018), only scattered reports exist on its biological functions in the periphery. Indeed, αS is ubiquitously expressed in non-neuronal tissues (Hashimoto et al., 1997; Askanas et al., 2000; Baltic et al., 2004; Nakai et al., 2007; Jiménez-Jiménez et al., 2023), including a variety of blood cell types (Pei and Maitta, 2019). In detail, erythrocytes possess exceedingly high concentrations of αS, representing a major source of αS in the periphery (Barbour et al., 2008; Tian et al., 2019). From erythrocytes, αS is released extracellularly to be endocytosed by circulating monocytes, thus concentration-dependently influencing their biology, phenotype, and functions (Liu et al., 2022). This is reminiscent of a “cell-to-cell” spread mechanism that is widely explored in the nervous and enteric systems (Schaeffer et al., 2020). Notably, while endogenous αS in periphery sustains normal innate and adaptive immune responses (Alam et al., 2022), abnormal levels of either exogenous or endogenous αS may promote abnormal immune reactions (Allen Reish and Standaert, 2015; White et al., 2018; Grozdanov et al., 2019; Grozdanov and Danzer, 2020; Limanaqi et al., 2024), including hyper-inflammation and functional alterations in monocytes and macrophages (Klegeris et al., 2008; Gardai et al., 2013; Haenseler et al., 2017; Pellegrini et al., 2022). This is key for the pathobiology of PD, since peripheral monocytes from these patients display a hyper-activated and pro-inflammatory profile (Su et al., 2022) and peripheral monocyte entry in the CNS has been postulated as a key event for αS-induced inflammation and neurodegeneration (Harms et al., 2018). In line with a bidirectional connection between the CNS and peripheral immune system (Natale et al., 2021), evidence in experimental models of PD suggests that neuron-released αS is taken up by macrophages, and in turn, excess/mutant αS is released from monocytes/macrophages and spreads to neurons, contributing to PD-like neuropathology (Moriya et al., 2022). Pathways that have been proposed to be involved in αS internalization either as a free protein, or through extracellular vesicles, include passive diffusion, and direct penetration through the plasma membrane, as well as conventional, and receptor-mediated endocytosis (Haenseler et al., 2017; Brás and Outeiro, 2021; Liu et al., 2022). These considerations suggest that, independently of the source, and spreading mechanisms, αS-induced biological alterations in peripheral cells might promiscuously impinge on the onset/progression of systemic inflammation and neurological diseases. However, the detailed cellular and molecular mechanisms through which αS influences monocyte and macrophage biology remain to be elucidated. αS is suggested to act as an alarmin that activates pro-inflammatory and stress-related pathways through a receptor-mediated mechanism mostly involving TLR4 and TLR2 (Fellner et al., 2013; Kim et al., 2013; Venezia et al., 2021). This notwithstanding, only scattered evidence exists on how intracellular αS, as well as its mobilization dynamics, and functional interactions contribute to shaping monocyte/macrophage biology and functions (Klegeris et al., 2008; Freeman et al., 2013; Gardai et al., 2013; Haenseler et al., 2017).

A key role of the autophagy pathway in governing proteostasis of either endogenous or exogenous αS is documented in several experimental models, including neurons, glia, and recently, T lymphocytes, and induced Pluripotent Stem Cell (iPSC)-derived macrophages (Martinez-Vicente et al., 2008; Colasanti et al., 2014; Haenseler et al., 2017; Ryskalin et al., 2018; Limanaqi et al., 2019; 2021b; Choi et al., 2020; Tang et al., 2021). While autophagy has been implicated in monocyte/macrophage function (Zhang et al., 2012; Germic et al., 2019), scarce evidence exists on whether/how αS affects autophagy in monocytes and macrophages specifically, and its functional consequences. In the light of a high affinity of αS for lipid-rich biological membranes, lipid metabolism also deserves to be investigated as a potential mechanism bridging the effects of αS, biogenesis/maturation of degradative organelles, and inflammatory status in macrophages (Shpilka and Elazar, 2015; Alza et al., 2019; Jarc and Petan, 2020). Based on these premises, herein we investigated the subcellular, molecular, and functional effects of excess αS in human THP-1 monocytic cell line, and THP-1-derived macrophages, and, at least preliminarily in blood-isolated monocyte-derived macrophages (MDMs). Results confirmed that extracellular αS is taken up by THP-1 monocytes, as well as THP-1-derived macrophages, and primary human MDMs. Despite inducing an apparently similar pro-inflammatory phenotype, the effects of excess αS in THP-1 cells, and THP-1-derived macrophages are associated with opposite outcomes on cell survival, and autophagy, along with functional alterations in migration/chemotaxis, and phagocytic capacity, respectively.

## 2 Materials and methods

### 2.1 Recombinant protein, cell culture, and treatments

#### 2.1.1 Recombinant protein

Recombinant human αS was purchased from Merck-Sigma (500 ug, S7820). The lyophilized protein was reconstituted in sterile PBS w/o calcium and magnesium at a final concentration of 1 mg/mL (70 μM) as per manufacturer instructions. 50 uL aliquots were prepared and stored at −20°C. A limulus amebocyte lysate (LAL) chromogenic kit (# HIT302**,** Hycult Biotech) with a minimum detection limit of 0.04 EU/mL was used to measure endotoxin levels before treatment. Endotoxin levels were <0.5 EU/mL and the protein was used at a final dilution of 14 µg/mL (1 μM concentration for 4 or 24 h) based on previous studies (Pellegrini et al., 2022; Limanaqi et al., 2024) and our pilot experiments.

#### 2.1.2 Cell cultures: THP-1 monocytic cell line, and differentiation to THP-1-derived macrophages

THP-1 cells (ATCC TIB-202, human acute monocytic leukemia cell line) were grown in RPM1 supplemented with 10% Fetal Bovine Serum (FBS, EuroClone, Milan, Italy), and 1% penicillin-streptomycin/L-glutamine (EuroClone, Milan, Italy) at 37°C in a humidified 5% CO^2^ atmosphere. Cells were maintained in 75 cm suspension flasks and passaged every 3 days when they reached 1 × 10^6^/mL concentration. Cells were routinely checked for mycoplasma contamination, and cell viability/count was determined using a Bio-Rad TC20 Automated Cell Counter (Bio-Rad Laboratories).

According to the different methodological assays, THP-1 cells were seeded in either 12-well (4 × 10^5^/well, in 1 mL), 24-well (2 × 10^5^/well, in 0.5 mL), or 96-well (5 × 10^4^/well, in 0.1 mL) suspension plates and cultured in RPMI 1640 with 10% of FBS (EuroClone, Milan, Italy). THP-1 cell medium was then supplemented with αS (1 uM), for 4 or 24 h. At these time-points, cells and supernatants were collected for different analyses and/or further experiments.

As a rough 30%–40% of cells are lost during and after the differentiation process from THP-1 cells to macrophages, for such experiments, THP-1 cells were seeded in excess compared to experiments with their undifferentiated counterpart in order to have a similar final number for both cell types. In detail, THP-1 cells were seeded in adherent 12-well plates (6 × 10^5^/well, in 1 mL), 24-well plates (3 × 10^5^/well, in 0.5 mL), or 96-well plates (7.5 × 10^4^/well, in 0.1 mL) in RPMI 1640 with 10% of FBS (EuroClone, Milan, Italy). For confocal microscopy, cells were seeded in u-Slide 8 Well high ibiTreat: #1.5 polymer coverslip chambers (CAT 80806, Twin Helix, Milan, Italy). Immediately after seeding, cells were treated with 25 ng/mL phorbol myristate acetate (PMA) (Sigma, St. Louis, MO) for 36 h based on previous studies showing that concentration of PMA for stable differentiation ranges from 5 to 100 ng/mL (Park et al., 2007). Cell differentiation was assessed by optical microscope observation (ZOE Fluorescent Cell Imager; Bio-Rad Laboratories). After washing in PBS to remove non-adherent/non-differentiated cells, THP-1-derived macrophages were incubated in fresh RPMI 1640 with 10% FBS for 4 h and then treated with αS (1 uM) for 4 and 24 h. Cells were then collected/fixed for further analyses.

#### 2.1.3 Cell cultures: primary human monocyte-derived macrophages

Thirty mL of whole blood were collected in EDTA-containing vacutainer tubes (Becton Dickinson, Rutherford, NJ) from 3 healthy volunteers. Peripheral blood mononuclear cells (PBMCs) were separated on lymphocyte separation medium (Cedarlane Laboratories, Hornby, ON, Canada) as previously described (Cui et al., 2021). Leukocyte viability was determined using a Bio-Rad TC20 Automated Cell Counter (Bio-Rad Laboratories). The percentage of CD14^+^ monocytes was determined in PBMCs by Flow cytometry as previously described (Saulle et al., 2021). Accordingly, a PBMC volume containing 2.5 × 10^5^ monocytes was seeded in 24-well plates in RPMI 1640 without FBS (EuroClone, Milan, Italy) and cultured for 2 h to isolate monocytes via plastic adherence. After washing in PBS, adherent monocytes were incubated with 100 ng/mL Macrophage Colony-Stimulating Factor (M-CSF, R&D Systems, Minneapolis, MN) in RPMI 1640 with 20% of FBS (EuroClone, Milan, Italy) added each 2 days for 5 days to generate monocyte-derived macrophages (MDMs) as previously described (Saulle et al., 2021). Differentiation was assessed by optical microscope observation (ZOE Fluorescent Cell Imager; Bio-Rad Laboratories). After washing in PBS, MDMs were incubated in fresh RPMI 1640 with 10% FBS, treated with αS for 24 h, and fixed for immunofluorescence.

### 2.2 Cell viability

THP-1 monocytic cell or PBMCs viability was determined through Trypan Blue exclusion assay and 3-(4,5-dimethylthiazol-2-yl)-2,5-diphenyltetrazolium bromide (MTT) assay. For Trypan Blue assay, 10 μL of cell suspension were mixed and briefly incubated with 10 μL of 0.4% Trypan Blue (Merck-Sigma, Milan, Italy) in 96-well plates. According to the manufacturer protocol, 10 μL of the mix were loaded on chamber slides and counted with the T20 Automated Cell Counter (Bio-Rad Laboratories, Hercules, CA, United States).

MTT was used to assess the viability of both THP-1 monocytic cells, and PMA-differentiated THP-1 macrophages (both untreated and αS-treated at 4 and 24 h) in previously seeded cells (96-well plates) and as previously described (Van Meerloo et al., 2011). Briefly, 30 μL of 3-(4,5-dimethylthiazol-2-yl)-2,5-diphenyltetrazolium bromide (MTT, final concentration 0.5 mg/mL) were added to each well under sterile conditions, and the 96-well plates were incubated for 4 h at 37°C. Supernatants were removed, and dimethyl sulfoxide (100 µL/well) was added. THP-1 cells were transferred into V-shaped 96-well plates and centrifuged to discard the supernatant. Cells were then resuspended in DMSO and transferred into flat 96-well plates. The plates were agitated on a shaker for 10 min and the absorbance of each well was measured at 490 nm with a Bio-Rad automated EIA analyzer (Bio-Rad Laboratories, Hercules, CA, United States). The viability of Control cells (Untreated) was arbitrarily considered as 100%, while the other conditions were expressed as percentage of control.

### 2.3 RNA extraction and transcriptional analyses through real-time qPCR

For transcriptional analyses, cells were resuspended/collected in RNAzol^®^ (TEL-TEST Inc., Friendswood, TX, United States) and RNA extraction was performed through the phenol-chloroform method, as previously described (Toni et al., 2018; Limanaqi et al., 2022). RNA was quantified by the Nanodrop 2000 Instrument (Thermo Scientific, Waltham, MA, United States). Briefly, 1 μg of RNA was purified from genomic DNA with RNase-free DNase (RQ1 DNase; Promega) and reverse transcribed into cDNA with Moloney murine leukemia virus reverse transcriptase along with random hexanucleotide primers, oligo dT, and dNTPs (Promega, Fitchburg, WI, United States). cDNA (25 ng) was amplified and quantified by real-time qPCR (CFX96 connect, Bio-Rad, Hercules, CA, United States) through the Universal SYBR^®^ Green Supermix (Bio-Rad, Hercules, CA, United States) in a final reaction mix volume of 10 uL. The following genes were analyzed: *Interleukin-1b (IL-1b), Interleukin-6 (IL-6), Chemokine CC-motif ligand 2 (CCL2), Chemokine CC-motif ligand 4 (CCL4), NOD-, LRR- and pyrin domain-containing protein 3 (NLRP3), Caspase-1 (CASP1), Nuclear Factor kappa B (NF-kB), Triggering Receptor Expressed in Myeloid Cells 1 (TREM1), Triggering Receptor Expressed in Myeloid Cells 1 (TREM2), Signal Transduction and Activator of Transcription 1 (STAT1), Tumor Necrosis Factor alpha (TNF-a), Sequestesome 1 (SQSTM/p62), Microtubule-Associated Protein 1A/1B Light Chain 3B (MAP1LC3B), Lysosomal-Associated Protein 1 (LAMP1), mammalian/mechanistic Target of Rapamycin (mTOR), Soluble N-ethylmaleimide-sensitive factor Attachment Protein 29 (SNAP29), B-cell lymphoma 2 (BCL-2), Bcl-2 Associated X-protein (BAX), Cholesterol 25-Hydroxylase (CH25H), ATP-binding cassette transporter G1 (ABCG1), Caveolin-1 (CAV-1), Liver X Receptor (LXR), X-box Binding Protein 1 (XBP1), Oxy-Sterol-Binding Protein (OSBP), Synuclein-alpha (SNCA), and Glyceraldehyde-3-Phosphate Dehydrogenase (GAPDH)*. All the primers were purchased as already optimized and used at 1x final dilution according to the manufacturer instructions (PrimePCR™ SYBR^®^ Green Assay, Bio-Rad). Reactions were performed according to the following thermal profile: initial denaturation (95°C, 15 min) followed by 40 cycles of 15 s at 95°C (denaturation) and 20 s at 60°C (annealing) and 20 s at 72°C (extension). Negative controls (distilled water, PCR mix non containing cDNA) were included in each run. Results for gene expression analyses were calculated by the 2^−ΔΔCT^ equation. Melting curves besides Ct values were analyzed for primer and reaction specificity. Results are presented as the mean N fold (percent) ± SEM of the relative expression units to an internal reference sample and normalized to the *GAPDH* housekeeping gene. Results show the quantifications from *n* = 3 independent experiments.

### 2.4 Multiplex elisa

The concentration of cytokines/chemokines was assessed in the supernatants of THP-1 cells and THP-1-derived macrophages in the presence/absence of αS by using precast immunoassays formatted on magnetic beads (Bio-Rad Laboratories, Hercules, CA, United States), according to manufacturer’s protocol via Luminex 100 technology (Luminex, Austin, TX). The concentrations of following cytokines were assessed: Interleukin-1b, -4 -5, −6, −7, −8, −10 (IL-1b, IL-4, IL-5, IL-6, IL-7, IL-8, IL-10), Granulocyte Colony-Stimulating Factor (G-CSF), Granulocyte-Macrophage Colony-Stimulating Factor (GM-CSF), Interferon Gamma (IFN-g), Monocyte Chemoattractant Protein 1/Chemokine CC-motif Ligand 2 (MCP1/CCL2), Macrophage Inflammatory Protein 1/Chemokine CC-motif Ligand 4 (MIP-1b/CCL4). Results are expressed as pg/mL from n = 3 independent biological replicates.

### 2.5 Flow cytometry

Detection of cell surface, and intracellular CCR2 was performed as previously described (Fujimura et al., 2015). In detail, after 4 or 24 h of αS incubation, THP-1 cells were harvested, centrifuged, and incubated with mouse anti-CCR2 monoclonal antibody (Beckman Coulter) in V-shaped 96-well plates (2.5 × 10^5^ cells/well/condition) to detect surface antigen for 15 min at RT, protected by light. Then, plates were centrifuged at 1,200 rpm for 8 min at RT to discard the antibody solution. Cells were washed in PBS (Euroclone, Italy), centrifuged again, and fixed in 1% paraformaldehyde (PFA, Sigma-Aldrich, MO, United States). For detection of intracellular antigen, cells were incubated with Intracellular Fixation & Permeabilization Buffer Set (eBioscience™, 88-8824-00) according to the manufacturer protocol, stained with mouse anti-CCR2 and/or anti-NLRP3 monoclonal antibody (Beckman Coulter) for 30 min at RT, and then washed with PBS and resuspended in 1% PFA. Acquisition was performed on a CytoFLEX™ flow cytometer system equipped with CytExpert software (Beckman Coulter), and data were analysed using Kaluza software, version 2.1.1. (Beckman Coulter). For CCR2, results are expressed as % of CCR2-expressing THP-1 cells (gating on total THP-1 cells), and Mean Fluorescence Intensity (MFI) of extra- and intra-cellular CCR2/total CCR2 MFI (gating on CCR2+ THP-1 cells) from n = 3 independent biological replicates. For NLRP3, results show the total MFI of NLRP3 in THP-1 cells from n = 3 independent biological replicates.

### 2.6 Transwell migration assay

Transwell Inserts (5.0-μm pores, Corning Costar 3,421) were used for migration/chemotaxis assay as previously described (Macanas-Pirard et al., 2017). Briefly, untreated, and αS-treated THP-1 cells (4 and 24 h) were harvested and pelleted by centrifuging at 1,200 rpm for 10 min at RT. Cells were resuspended in fresh RPMI with 5% FBS, counted and seeded on the upper chamber of the 24-well Transwell plate (1.5  ×  10^5^/well). The conditioned media (supernatants) recovered from both 4 h and 24 h, CTR untreated and αS-treated THP-1 cells containing 10% FBS, along with different concentrations of cytokines and chemokines previously assessed through Multiplex ELISA, were applied in lower chambers for chemotaxis assay. A condition with RPMI medium with 10% FBS (unconditioned medium) in lower chamber was included as the spontaneous migration control/negative control of chemotaxis. Thus, cells from each experimental condition (Untreated, and αS-treated, 4 and 24 h) were co-cultured with i) RPMI + 10% FBS medium, ii) conditioned medium from untreated THP-1 cells containing RPMI + 10% FBS + low/negligible amount of cytokines/chemokines, and iii) conditioned medium from αS-treated THP-1 cells containing RPMI + 10% FBS enriched in cytokines/chemokines. After 16 h, inserts were removed and migrated cells were i) resuspended and counted thrice with the T20 Automated Cell Counter (Bio-Rad Laboratories, Hercules, CA, United States), and ii) left to settle down and imaged at optical microscope (ZOE Fluorescent Cell Imager; Bio-Rad Laboratories, Hercules, CA, United States). The number of migrated cells is shown as mean percentage (calculated out of the total cells seeded in the upper chamber) ± SEM (*n* = 3 biological replicates).

### 2.7 Western blot

Cells were lysed in ice-cold RIPA lysis buffer supplemented with 2% SDS, and a cocktail of protease and phosphatase inhibitors (cOmplete and PhosSTOP; Roche Applied Science, Mannheim, Germany), sonicated, incubated on ice for 30 min on a platform rotator, and then centrifuged at maximum speed for 30 min at 4°C. Protein concentration from whole cell lysates was determined through BCA protein assay kit (Pierce, United States). 30-to-40 μg proteins per sample were prepared by combining the appropriate volume with 4 ×Reducing Laemmli SDS sample buffer (final 1×, J60015.AC, Thermo-Fisher Scientific) and Milli-Q H_2_O. Samples were boiled for 5 min and loaded on 4%-12% Mini-Protean Stain-Free TGX Precast SDS-PAGE gel (4,568,095, Bio-rad, Hercules, CA, United States), which was run at 100 V for 1.5 h. The gel was activated at ChemiDoc MP imaging system (Bio-Rad, Hercules, CA, United States) and proteins were transferred on PVDF membrane through The Trans-Blot Turbo Transfer System TM and Transfer Pack TM (Bio-Rad, Hercules, CA, United States). For αS immunodetection and preservation of protein, membrane was fixed with 0.4% PFA for 30 min at RT as previously described (Stojkovska and Mazzulli, 2021; Limanaqi et al., 2024). The stain-free membrane was imaged at ChemiDoc MP imaging system (Bio-Rad, Hercules, CA, United States) and then blocked in 1×TBS with 5% Bovine Serum Albumin (BSA) for 1 h at RT, washed in TBS-0.1% Tween, and incubated overnight at 4°C with the following antibodies prepared in 2.5% BSA/non-fat dry milk: rabbit anti-alpha-synuclein antibody (1:800, Bioss, United States, BSM-54277R 3H12), rabbit anti-LC3B (1:1000, Cell Signaling, #3868), mouse anti-SQSTM1/p62 (1:1000, Cell Signaling, #88588), rabbit anti-phosphorylated ribosomal protein subunit S6 (pS6, 1:1000, Cell Signaling, #4858), mouse anti-total ribosomal protein subunit S6 (S6, 1:1000 Cell Signaling, #2317), mouse anti-LAMP1 (1:1000, BD Biosciences, AB_398356), and mouse anti-β-actin (1:1000, Merck Sigma, A5316). The day after, the blot was washed 3 × 5 min with 1×TBS-0.1% Tween and incubated for 1 h with HRP-conjugated secondary antibodies goat anti-rabbit (1:5000, STAR208P, Bio-Rad) or goat anti-mouse (1:5000, BD Biosciences, HAF007) in blocking buffer with 2.5% BSA/non-fat dry milk. The blot was washed 3 × 5 min with 1×TBS-0.1% Tween and then incubated for 5 min with Clarity Western ECL substrate and visualized with a ChemiDoc MP imaging system (Bio-Rad, Hercules, CA, United States). For membrane reprobing, a stripping solution (25 mM glycine-HCl, pH 2, supplemented with 1% SDS) was used for approximately 30 min under constant agitation, followed by 3 × 5 min washes in 1×TBS-0.1% Tween and 1 h incubation in blocking solution. Results were analyzed using the Image Lab software (Bio-Rad, Hercules, CA, United States). Quantification was performed through normalization to β-actin. For each experiment, a representative blot is shown, and the graphs show to the mean ± SEM from n = 3 independent experiments.

### 2.8 Immunofluorescence at confocal microscopy

For immunofluorescence, we followed previously described protocols (Zecchini et al., 2019). Cells cultured in u-Slide chambers and stimulated as described above, were fixed in 4% paraformaldehyde (PFA) for 15 min and permeabilized with 0.1% Triton 100X for 10 min. After brief washing in PBS, cells were incubated in a blocking solution of 5% BSA for 1 h, and then incubated at for 1 h at RT with the following primary antibodies prepared in 1% BSA: rabbit anti-α-syn (1:200, Bioss, United States, BSM-54277R 3H12), mouse anti-SQSTM1(1:1000, Cell Signaling, #88588), rabbit anti LC3B (1:1000, Cell Signaling, #3868), or mouse anti-LAMP1 (1:1000, BD Bioscences, AB_398356). Cells were washed thrice in PBS and incubated with Alexa-Fluor-conjugated secondary antibodies raised against the host species of the primary antibodies, namely Goat anti-mouse Alexa Fluor 488 (Abcam, ab150113) or 647 (Abcam, ab150115), or Goat anti-rabbit Alexa Fluor 488 (Abcam, ab150077) or 647 (Abcam, ab150079), 1:500 prepared in 1% BSA-PBS (Prodotti Gianni, Milan, Italy). Negative controls were performed by omitting primary antibodies. For F-actin and/or nuclear counterstaining, respectively, Phalloidin (Alexa Fluor™ 647 Cat A22287 or Alexa Fluor™ 594 Cat A12381, Thermo-Fisher Scientific, final concentration 1:100) and/or DAPI (Invitrogen ThermoFisher Scientific, D1306, final concentration 1:1000) were added to the secondary antibody solution, followed by 3 × 5 min washes in PBS. For LysoTracker-based lysosomal staining, 30 min prior to fixation, cells were incubated with 50 nM LysoTracker^®^ Red DND-99 (Cat L7528, Thermo-Fisher Scientific). For examination of lipid droplets by Oil red O stain (Merck Sigma O0625) together with immunolabelled cellular constituents we used a protocol which was slightly modified form Koopman et al. (Koopman et al., 2001). Briefly, Oil red O was dissolved to a stock solution by adding 500 mg Oil red O to 100 mL isopropanol. Prior to staining, a 36% working solution, containing 12 mL Oil red O stock solution and 8 mL deionised water was prepared and 0.22 uM filtered. After fixation, cells were briefly washed in 60% isopropanol, immersed in the working solution of Oil red O for 30 min, and then rinsed thrice with deionised water. Immune-staining was then performed as routinely. u-Slides were *in situ* mounted by adding Fluoromount medium (F4680, Merck-Sigma, Milan, Italy). Confocal images were acquired on a TCS SP8 System equipped with a DMi8 inverted microscope and a HC PL APO 40×/1.30 Oil CS2 (Leica Microsystems, Wetzlar, Germany) at a resolution of 1024 × 1024 pixels. Image analyses (Integrated density or count of puncta/cells) were performed through ImageJ software (NIH, Bethesda, MD, United States). Quantifications were performed from at least n = 3 microscopy fields per experimental group, and per each independent experiment.

### 2.9 Phagocytosis assay

THP-1–derived macrophages previously cultured in u-Slide chambers and stimulated as described above were incubated for 2 h with latex beads coated with rabbit IgG–FITC complex (No. 500290, Cayman Chemical, final dilution 1:100) at 37°C in a humidified 5% CO2 atmosphere, according to the manufacturer protocol, and as previously described (Mysore et al., 2021). Beads were added 2 h before the ending of αS incubation. Cells were gently washed twice with pre-warmed Assay buffer prepared in sterile Milli-Q water (No. 500290, Cayman Chemical) to remove non-phagocytosed beads. Cells were then either i) immediately fixed in 4% PFA, or ii) replenished with fresh medium and incubated at 37°C, 5% CO2 for additional 20 h to monitor the intracellular fate of phagocytosed beads/FITC before fixation. In both cases, 30 min prior to fixation, cells were incubated with 50 nM LysoTracker^®^ Red DND-99 (Cat L7528, Thermo-Fisher Scientific). Fixed cells were then stained with Alexa Fluor™ 647 Phalloidin for 30 min (Cat A22287 Thermo-Fisher Scientific, final concentration 1:100), washed twice in PBS and imaged. Confocal images were acquired on a TCS SP8 System equipped with a DMi8 inverted microscope and a HC PL APO 40×/1.30 Oil CS2 (Leica Microsystems, Wetzlar, Germany) at a resolution of 1024 × 1024 pixels. Phagocytosed cargo (FITC), and acidic organelles (LysosoTracker) were quantified and expressed as percentage of cell area/phalloidin, while co-localization of phagocytosed cargo and lysosomes was calculated through the Pearson’s R coefficient through the ImageJ software (NIH, Bethesda, MD, United States). Results are expressed as mean ± SEM from n = 3 independent biological replicates.

### 2.10 Statistical analysis

The GraphPad Prism software package (GraphPad Software, San Diego, CA, United States) was used to generate all the graphs and perform statistical analyses. Data normality was assessed through the Shapiro–Wilk test. The statistical significance was tested through one-way, or two-way ANOVA, followed by the Fisher’s LSD post-hoc test. Statistically significant *p* values are shown in the graphs as *p < 0.05, **p < 0.01, ***p < 0.001, ****p < 0.0001. To avoid graphs overcrowding, *p* values are shown for statistically significant groups only. Results are expressed as mean ± SEM from n = 3 independent experiments/biological replicates.

## 3 Results

### 3.1 THP-1 cells take up exogenous αS in the absence of cell toxicity

**FIGURE 1:**
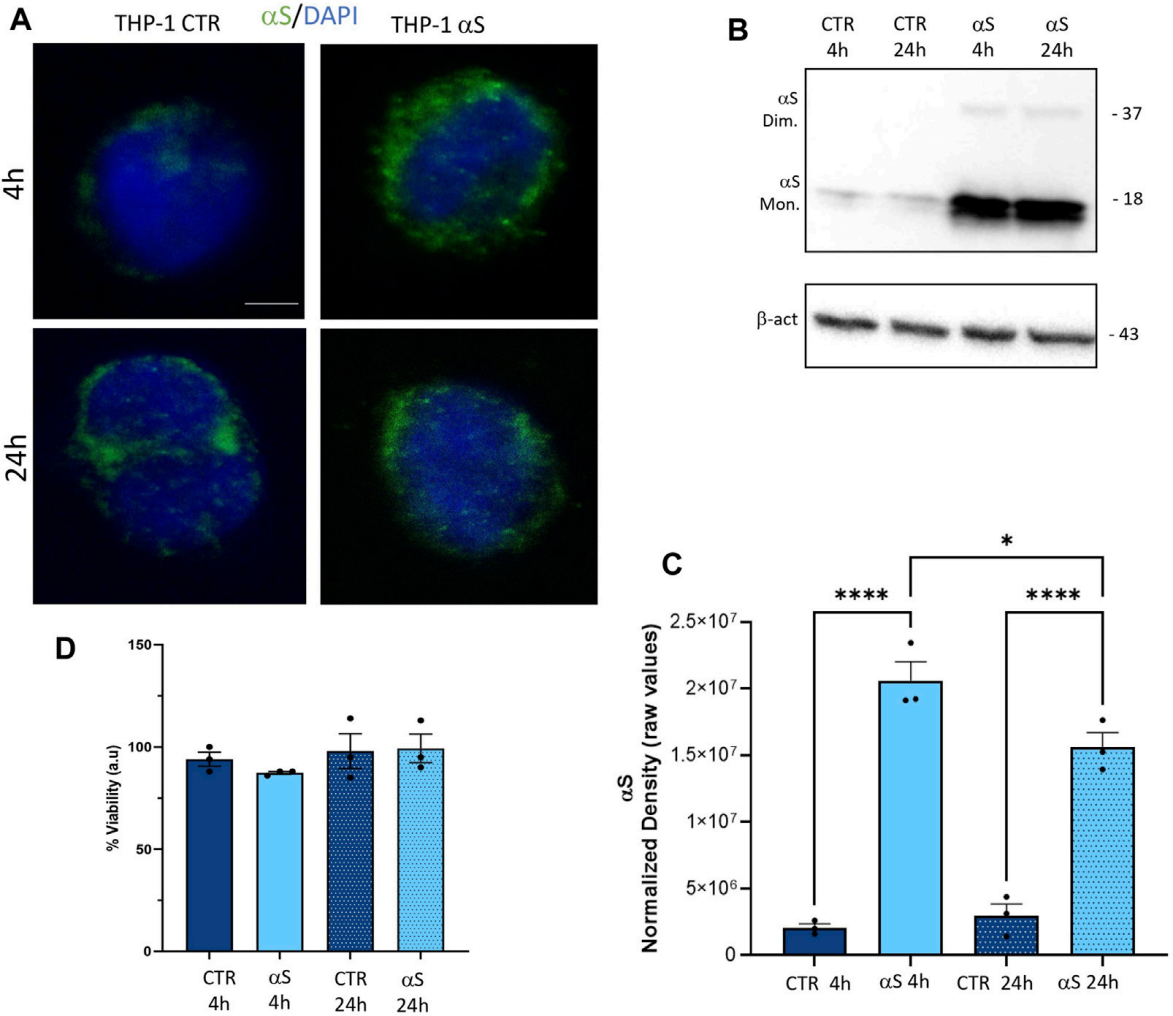
THP-1 cells take up exogenous αS in the absence of cell toxicity **(A)**. Representative Immunofluorescence for t αS in control (CTR) and αS-exposed THP-1 cells for 4 h and 24 h. Bars correspond to 5 μm **(B)**. Representative Western blot for αS and β-actin in control (CTR) and αS-exposed THP-1 cells for 4 h and 24 h **(C)**. Quantification of monomeric αS normalized to β-actin. Results are shown as normalized raw values corresponding to mean ± SEM. ***p < 0.001; ****p < 0.0001 **(D)**. The graph shows results from MTT assay in control (CTR) and αS-exposed THP-1 cells for 4 h and 24 h. Results are shown as arbitrary units (a.u) corresponding to mean ± SEM.

Both immunofluorescence (Figure 1A), and western-blot analysis (Figure 1B) showed that control THP-1 cells express low constitutive levels of αS (CTR), and they effectively internalize exogenous αS following 4 and 24 h exposure. Intracellular exogenous αS detected at 4 and 24 h post-exposure corresponded predominantly to monomeric species, through faint dimers could be observed as well (Figure 1B). Interestingly, the amount of quantified αS at 24 h was moderately, though significantly lower (roughly by 27%) compared to that detected at 4 h (Figure 1C), suggesting that THP-1 cells are able to dilute and probably degrade the intracellular cargo of excess αS. In line with this, 1 μM αS did not cause toxicity in THP-1 cells neither at 4 nor at 24 h (Figure 1D).

### 3.2 Characterization of αS-induced inflammatory profile in THP-1 cells

**FIGURE 2:**
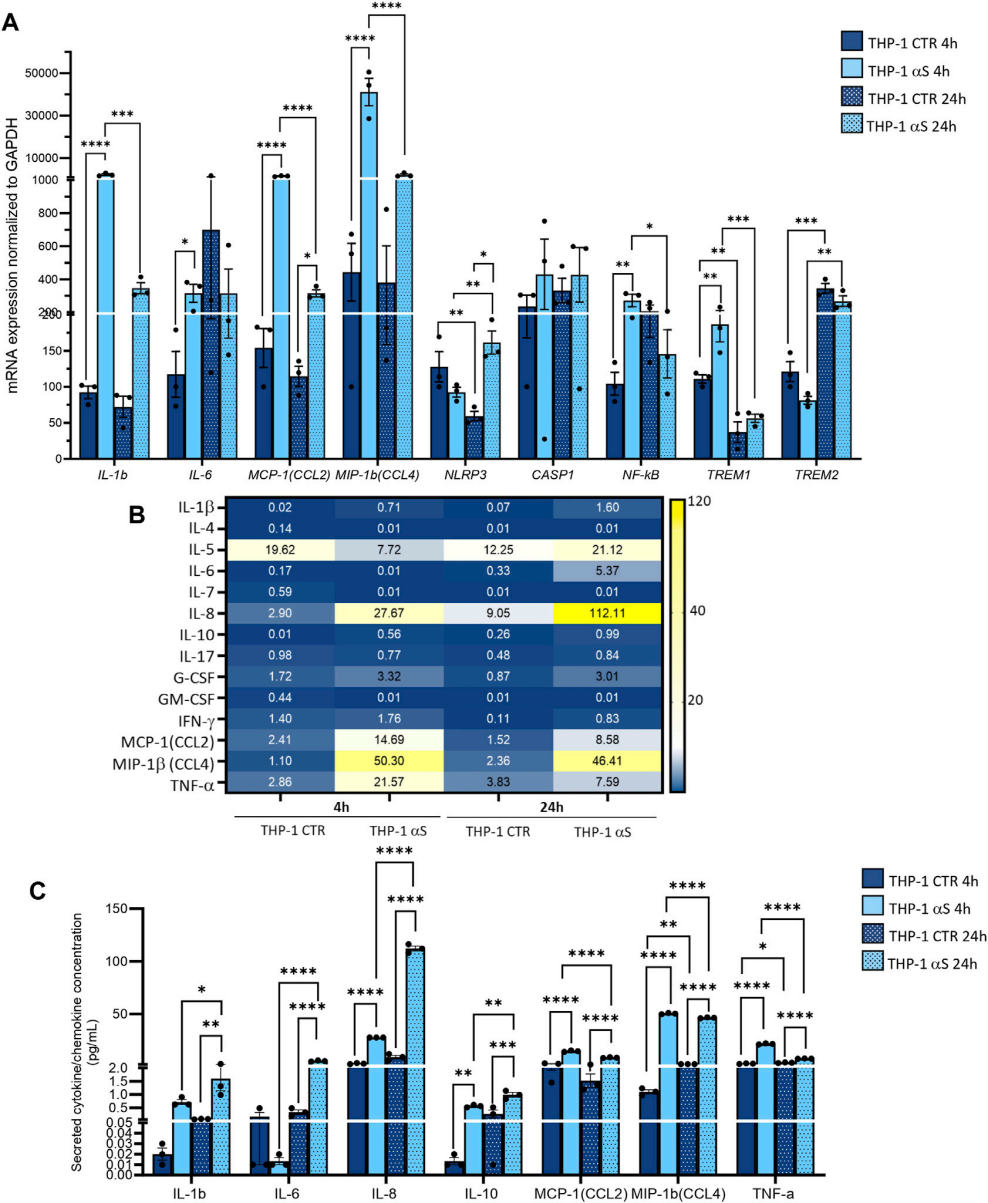
Characterization of the inflammatory profile in THP-1 cells. **(A)** Real time RT-qPCR analysis of inflammatory genes in control (CTR) and αS-exposed THP-1 cells (4 h and 24 h). Values were normalized to *GAPDH* and are shown as mean ± SEM. **(B)** Heatmap showing overall mean concentrations (pg/mL) of secreted cytokines/chemokines measured by Multiplex Elisa from the supernatants of CTR, and αS-exposed THP-1 cells (4 h and 24 h). **(C)** Graph showing only statistically significant secreted cytokines/chemokines measured through Multiplex Elisa from the supernatants of CTR, and αS-exposed THP-1 cells (4 h and 24 h). Results are shown as mean ± SEM. *p < 0.05; **p < 0.01; ***p < 0.001; ****p < 0.0001.

Both intracellular mRNA expression and concentrations of secreted cytokines/chemokines were quantified in CTR, and αS-exposed THP-1 cells. αS exposure at either 4 or 24 h produced a general transcriptional upregulation of genes encoding pro-inflammatory cytokines/chemokines/mediators (Figure 2A). However, different profiles were observed at 4 h and 24 h αS exposure. In detail, the mRNA expression of IL-1β, IL-6, MCP-1/CCL2, MIP-1β/CCL4, NF-kB, and TREM1 was significantly higher in 4 h αS compared with 4 h CTR cells. At 24 h, such an upward trend induced by αS was reproduced for MCP-1/CCL2, MIP-1β/CCL4, and NLRP3 mRNA expression (Figure 2A). Conversely, the expression of IL-1β, MCP-1/CCL2, MIP-1β/CCL4, NF-kB, and TREM1 was significantly reduced, whereas that of TREM2 was upregulated in 24 h compared to 4 h αS-exposed THP-1 cells (Figure 2A). Notably, despite upregulating NLRP3 mRNA expression at 24 h, αS did not affect the intracellular amount of NLRP3 inflammasome, as assessed through flow cytometry (Supplementary Figure S1).

αS exposure at either 4 or 24 h also produced a general increase in the concentration of secreted inflammatory cytokines/chemokines (Figure 2B). In detail, 4 h αS exposure significantly increased IL-8, IL-10, MCP-1/CCL2, MIP-1b/CCL4, and TNF-α compared with 4 h CTR THP-1 cells (Figure 2C). Such an upward trend was observed as well in 24 h αS exposure, as the concentration of secreted IL-1β, IL-6, IL-8, IL-10, MCP-1/CCL2, MIP-1β/CCL4, and TNF-α was significantly increased compared with 24 h CTR. Remarkably, while the concentration of IL-1β, IL-6, IL-8, and IL-10 was higher in 24 h-compared with 4 h-exposed αS, that of MCP-1/CCL2, MIP-1b/CCL4, and TNF-α was instead modestly, but significantly lower in 24 h-compared with 4 h-exposed αS (Figure 2C). These data suggest that αS induces a pro-inflammatory profile in THP-1 cells; however, the uncoupling between mRNA and intracellular/secreted cytokine/chemokines, and mostly, the differences observed between 4 h vs. 24 h αS exposure, suggest the occurrence of compensatory cell mechanisms balancing mRNA transcription, protein translation, turnover, and secretion of specific pro-inflammatory mediators.

We next investigated whether the peculiar inflammatory profile of αS-exposed THP-1 cells is associated with altered extracellular and intracellular dynamics of the MCP-1/CCL2 chemokine receptor CCR2. Initial results on flow cytometry showed that 4 h αS exposure significantly decreases the percentage of total THP-1 cells staining for CCR2 on the cell surface compared with CTR THP-1 4 h monocytes (Supplementary Figure S2A). When gating on CCR2-positive instead of total THP-1 cells, results showed that 4 h αS significantly decreased the mean fluorescence intensity (MFI) ratio of extracellular/total CCR2, meanwhile increasing that of intracellular/total CCR2 (Supplementary Figure S2B). Both analyses showed this effect to be specific to the 4 h αS exposure condition, as no differences were observed between 24 h αS-exposed and 24 h CTR THP-1 cells Supplementary Figure S2A, B). These results suggest that by potentiating CCL2 secretion, αS leads to rapid CCR2 activation followed by receptor internalization and desensitization, which peaks already following brief (4 h) exposure, while prolonged presence of αS (24 h) fosters CCR2 recycling back to the plasma membrane.

### 3.3 αS time-dependently alters migration and/or chemotaxis

**FIGURE 3:**
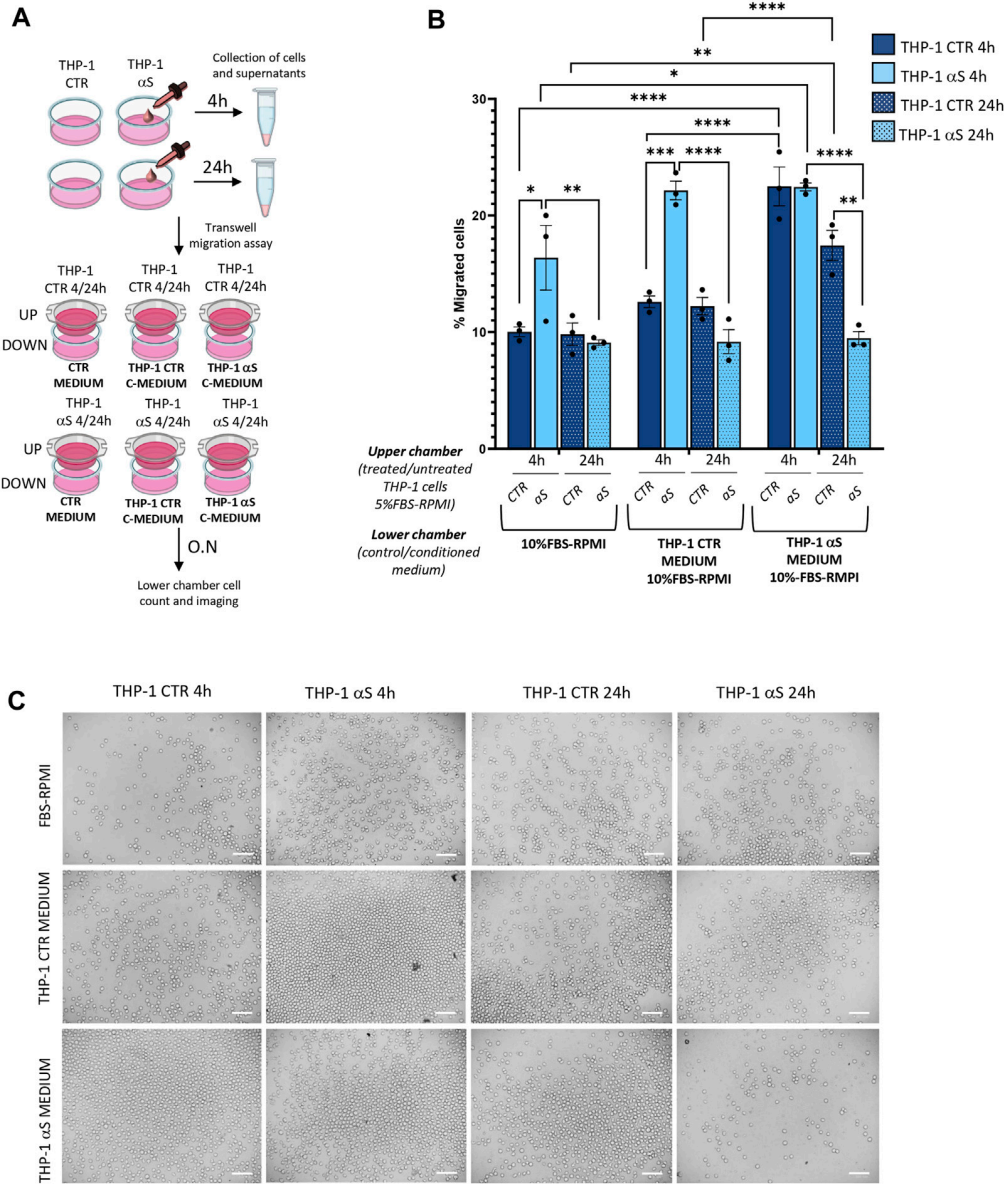
αS alters migration/chemotaxis of THP-1 cells. **(A)** Cartoon summarizing the set-up of the transwell assay with the different combinations of untreated (CTR)/αS-exposed cells in the upper chambers, and their unconditioned (CTR medium) or conditioned media/supernatants (C-Medium) in the lower chamber. **(B)** Count of migrated cells in the lower chambers of the transwell assay under different experimental conditions/combinations. Cells were counted through the Trypan Blue exclusion assay and were expressed as percentage (number of migrated cells out of the total number of cells seeded in the upper chamber). Values are shown as mean ± SEM. *p < 0.05; **p < 0.01; ***p < 0.001; ****p < 0.0001. **(C)**. Representative microscopy field of the migrated cells from the different experimental conditions/combinations. Bars correspond to 100 μM.

We next assessed whether αS-induced pro-inflammatory profile is associated with functional alterations in THP-1 cells, namely migration and chemotaxis. By combining treated/untreated cells, and unconditioned/conditioned media in a transwell assay model (Figure 3A), we assessed whether i) conditioned media (supernatants) from αS-exposed THP-1 cells (enriched in cytokines/chemokines as previously assessed) attract CTR THP-1 cells, and ii) αS exposure for 4 and 24 h alters THP-1 cells’ spontaneous migration and chemotaxis. Results showed that pooled conditioned media (supernatants) from 4 h to 24 h-αS-exposed THP-1 cells potently attract untreated CTR cells cultured for both 4 h and 24 h (Figure 3B, C). In detail, a higher percentage of CTR THP-1 cells migrated in the lower chamber containing conditioned medium (supernatants) collected from αS-exposed cells compared to THP-1 cells that migrated towards both i) the unconditioned medium with FBS, and ii) their own conditioned medium (supernatants that were pooled from 4 h to 24 h CTR THP-1 cells). Instead, increased migration of 4 h αS-exposed compared with CTR THP-1 cells was detected only in the presence of unconditioned medium containing FBS, and supernatant media from THP-1 CTR cells, suggesting spontaneous migration (Figure 3B, C). In the presence of chemokine-rich supernatants from αS-exposed cells, no differences were observed in terms of migrated cells between 4 h αS-exposed and CTR THP-1 cells, suggesting that 4 h αS-overloaded THP-1 cells do not feature increased chemotaxis (Figure 3B, C). Remarkably, migration/chemotaxis of THP-1 cells exposed to αS for 24 h was reduced compared with both 4 h αS-exposed cells, and 24 h CTR THP-1 cells (Figure 3B, C). In fact, a lower percentage of THP-1 cells exposed to αS for 24 h migrated towards their own chemokine-conditioned media compared to both untreated CTR 24 h, and their 4 h αS-exposed counterparts. Collectively, these data suggest that i) the extracellular milieu produced from αS-exposed cells serves as a chemoattractant for untreated monocytic cells, and ii) αS-loaded THP-1 monocytic cells feature altered migration capacity, and chemotaxis, with the effects varying according to the timing of αS exposure.

### 3.4 αS upregulates autophagy markers in THP-1 cells

**FIGURE 4:**
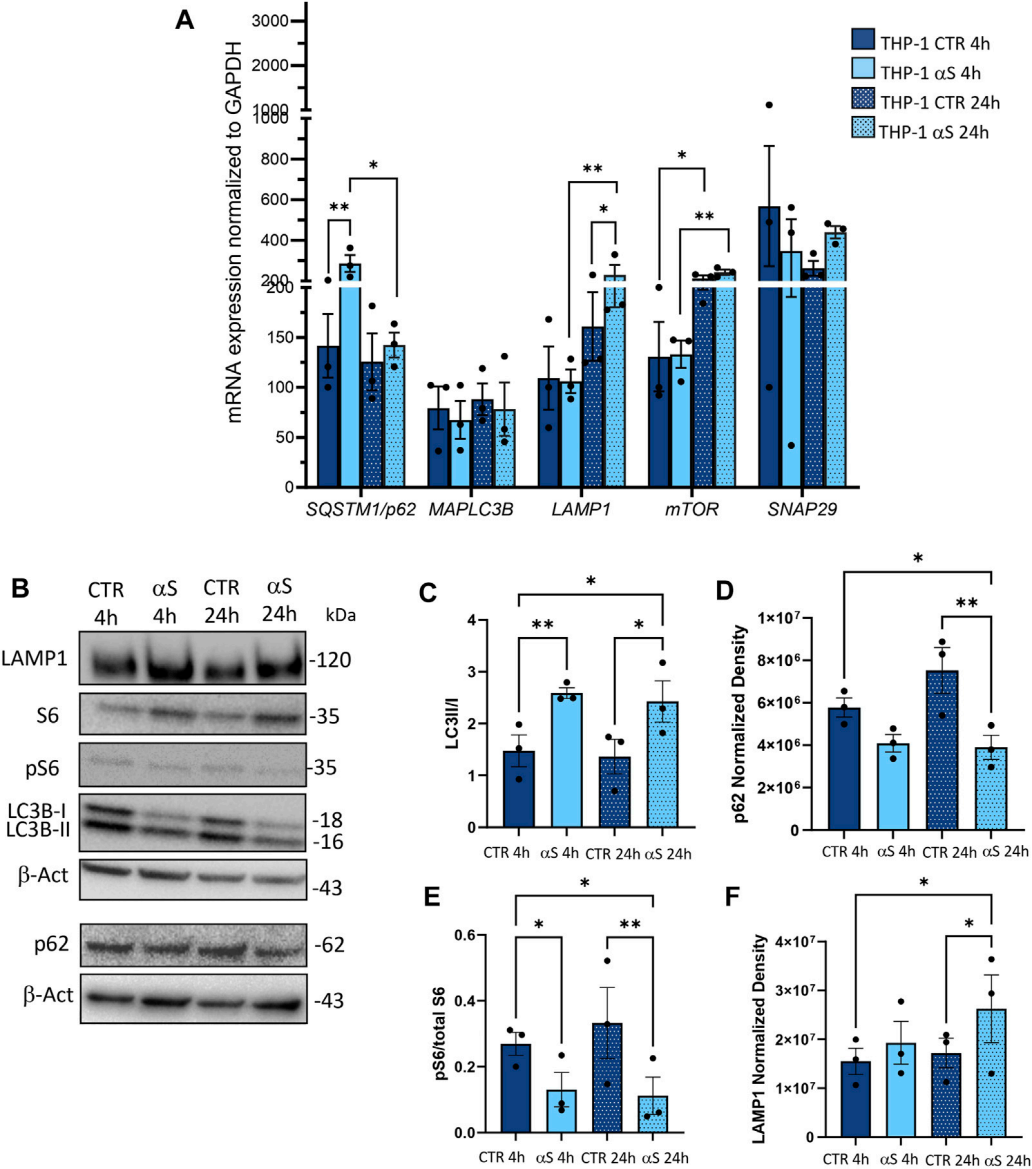
αS increases autophagy-lysosomal markers in THP-1 monocytic cells. **(A)** Real time RT-qPCR analysis of autophagy-related genes in control (CTR) and αS-exposed THP-1 cells (4 h and 24 h). Values were normalized to *GAPDH* and are shown as mean ± SEM. **(B)** Representative Western Blot against LC3II, LC3I, pS6, S6, p62, LAMP1 and β-actin in control (CTR) and αS-exposed THP-1 monocytes (4h and 24 h). **(C–F)** Quantification of LC3II/I ratio **(C)**, p62 **(D)**, LAMP1 **(E)**, and pS6/S6 ratio **(F)**. Normalization was performed against β-actin, and results are shown as normalized raw values corresponding to mean ± SEM. *p < 0.05; **p < 0.01.

To assess whether the effects of αS in THP-1 cells are associated with potential alterations in the autophagy/lysosomal pathway, we examined both mRNA expression (Figure 4A) and protein levels of autophagy-related markers (Figure 4B–F). Early αS exposure (4 h) upregulated mRNA expression of the autophagy adaptor SQSTM1/p62, while 24 h αS exposure significantly upregulated LAMP1 mRNA expression (Figure 4A). While SQSTM1/p62 mRNA expression was lower in 24 h compared to 4 h αS-exposed THP-1 cells, LAMP1 followed an opposite trend (Figure 4A). mTOR expression was increased in 24 h compared to 4 h in both CTR and αS conditions, yet it was not modified by αS exposure compared with CTR at either time-points. Likewise, αS exposure at either time-points did not significantly affect the mRNA expression of MAP1LC3B or SNAP29, which is known to promote autophagosome-lysosome fusion. Western Blot analysis showed that while decreasing overall LC3-II levels (data not shown), αS significantly increased LC3II/I ratio compared to CTR THP-1 cells both at 4 and 24 h, suggesting increased availability of the lipidated LC3 isoform for autophagosome formation (Figure 4C). This was parallelled by a decrease in p62 levels at 24 h αS exposure compared to CTR, suggesting effective degradation of p62 protein despite its early transcriptional upregulation (Figure 4D). In fact, while the early upregulation of SQSTM1/p62 mRNA expression induced by 4 h αS suggests an attempt to activate degradation pathways by increasing the availability of the autophagy adaptor protein (Figure 4A), the decrease in p62 protein levels by 24 h αS exposure suggests an effective progression of autophagy (Figure 4D). Supporting this claim, the pS6/total S6 protein ratio, a reliable index of mTOR activity, was significantly reduced by αS exposure at both time points compared with CTR THP-1 cells (Figure 4E). These data also suggest that pS6/S6 ratio is partly uncoupled to mTOR mRNA expression, which was not significantly modified by αS at either time-points. Instead, LAMP1 protein increase occurred at 24 αS exposure (Figure 4F), consistently with the transcriptional upregulation of LAMP1 mRNA by 24 h αS. Altogether, these data suggest that αS, mostly at 24 h exposure, elicits upregulation of autophagy/lysosomal markers, possibly via mTOR inactivation.

### 3.5 Characterization of αS-induced inflammatory profile and toxicity in THP-1-derived macrophages

**FIGURE 5:**
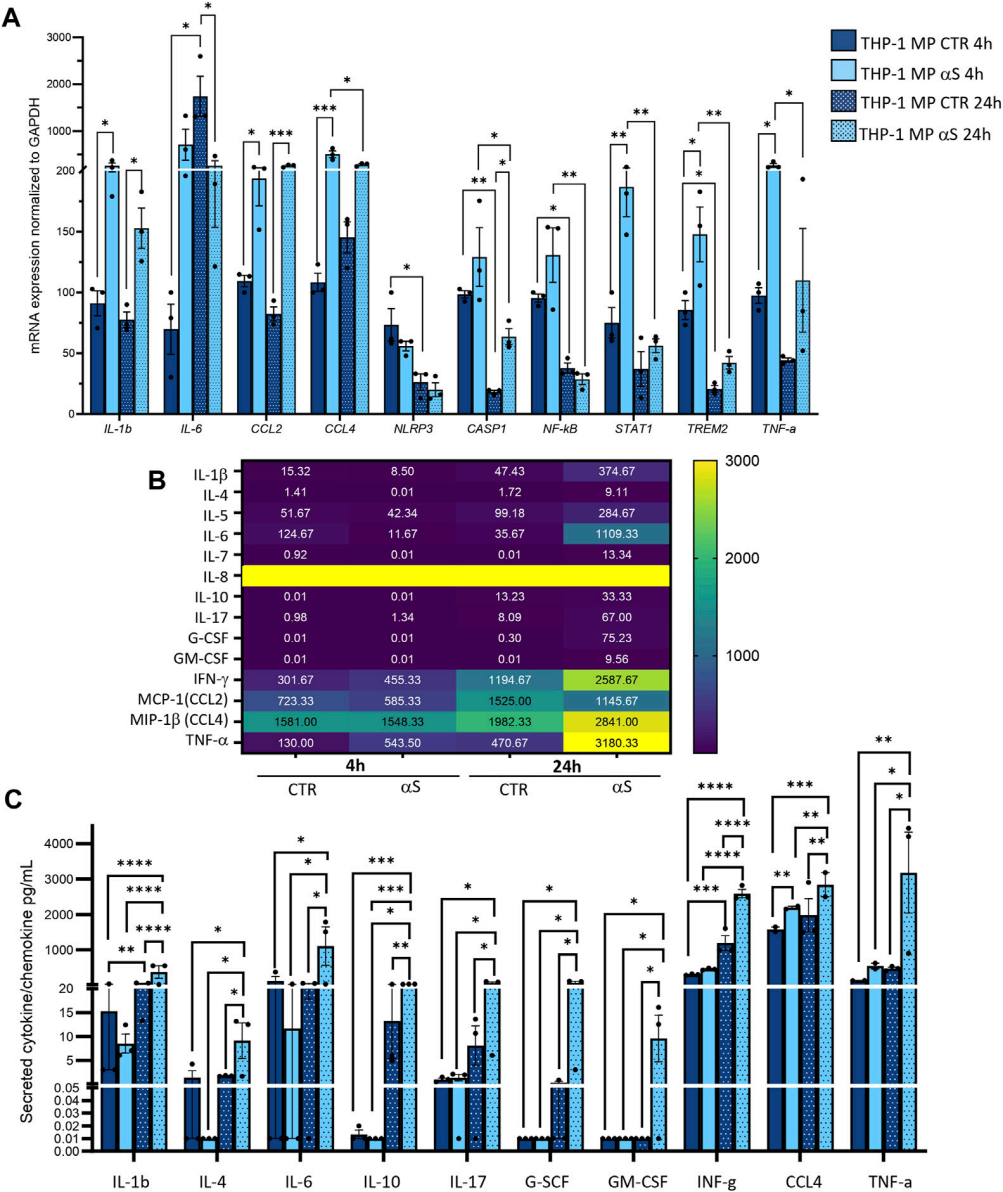
Characterization of αS-induced inflammatory phenotype in THP-1-derived macrophages. **(A)** Real time RT-qPCR analysis of inflammatory genes in control (CTR) and αS-exposed THP-1-derived macrophages (MP, 4 h and 24 h). Values were normalized to *GAPDH* and are shown as mean ± SEM. **(B)** Heatmap showing overall mean concentrations (pg/mL) of secreted cytokines/chemokines measured by Multiplex Elisa from the supernatants of CTR, and αS-exposed THP-1-derived macrophages (MP, 4h and 24 h). IL-8 concentrations were constantly above the detection limit. **(C)** Graph showing only statistically significant secreted cytokines/chemokines measured through Multiplex Elisa from the supernatants of CTR, and αS-exposed THP-1-derived macrophages (MP, 4 h and 24 h). Results are shown as mean ± SEM. *p < 0.05; **p < 0.01; ***p < 0.001; ****p < 0.0001.

Inflammatory profiles of CTR and αS-exposed THP-1-derived-macrophages were characterized similarly to their monocytic counterpart. In detail, mRNA expression of IL-1β, MCP-1/CCL2, MIP-1β/CCL4, STAT1, and TNF-α was significantly higher in 4 h αS compared with 4 h CTR THP-1-derived macrophages (Figure 5A). This upward trend persisted at 24 h αS exposure for IL-1β, MCP-1/CCL2, and MIP-1β/CCL4, along with upregulation of CASP1 mRNA expression (Figure 5A). Instead, IL-6 mRNA expression decreased in 24 h αS-exposed compared with CTR cells (Figure 5A). Again, the mRNA expression of CCL4, CASP1, NF-kB, STAT1, TREM1, and TNF-α was lower in 24 h compared with 4 h αS exposure (Figure 5A).

Contrary to what was observed for monocytic THP-1cells, αS exposure in THP-1-derived macrophages produced a time-related increase in the concentration of secreted inflammatory cytokines/chemokines (Figure 5B, C), except for CCL2 which showed a downward, though non statistically significant trend in 24 h vs. 4 h αS (Figure 5B). Instead, IL-8 concentrations were constantly above the detection limit (Figure 5B). In detail, significant differences between αS and CTR cells were observed for most secreted cytokines/chemokines only at 24h, when αS exposure significantly increased IL-1β, IL-4, IL-6, IL-10, IL-17, G-CSF, GM-CSF, IFN-γ, MIP-1b/CCL4, and TNF-α compared with CTR THP-1-derived macrophages (Figure 5C). The concentration of these very same cytokines/chemokines was also significantly higher in 24 h αS- compared with 4 h αS-exposed cells. This suggests that several mRNA reductions, including IL-6 expression at 24 h αS-exposed vs. CTR cells, as well as CCL4, and TNF-α expressions at 24 h compared with 4 h αS (Figure 5A), represent a compensatory response to increased cytokine production. Thus, αS potentiates the pro-inflammatory profile in THP-1-derived macrophages, which is magnified at 24 h αS exposure in terms of secreted cytokine/chemokines.

**FIGURE 6:**
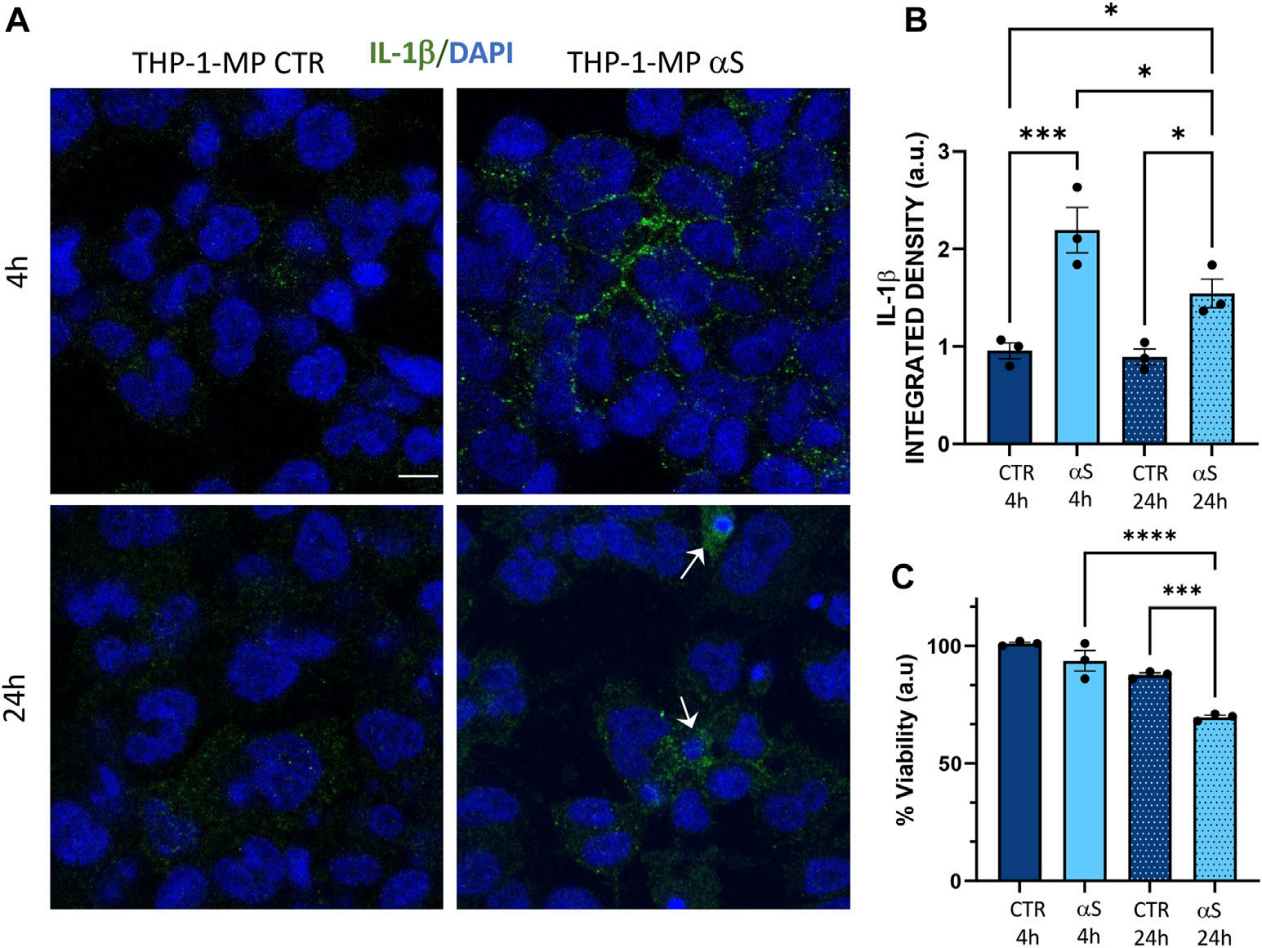
αS induces early intracellular accumulation of IL-1β in THP-1-derived macrophages. **(A)** Representative Immunofluorescence against IL-1β in control (CTR) and αS-exposed THP-1-derived macrophages (MP) for 4 h and 24 h. Arrows point at apoptotic/dying cells. Bars correspond to 10 μm. **(B)** Quantification of IL-1β integrated fluorescence density from control (CTR) and αS-exposed THP-1-derived macrophages (MP) for 4h and 24 h. Results are shown as mean ± SEM. *p < 0.05; ***p < 0.001; **(C)** MTT assay in control (CTR) and αS-exposed THP-1-derived macrophages for 4h and 24 h. Results are shown as arbitrary units (a.u) corresponding to mean ± SEM. ***p < 0.001; ****p < 0.0001.

Intracellular staining for IL-1β suggested that the lack of significant effects at 4 h αS in terms of secreted inflammatory mediators is due to the intracellular accumulation of this cytokine which peaks at this time-point (Figure 6A, B). In detail, intracellular IL-1β staining was significantly increased by both 4 h and 24 h αS exposure compared to CTR THP-1-derived macrophages; however, it was significantly lower in 24 h compared with 4 h αS exposure (Figure 6B), a trend which was opposite to that observed for secreted IL-1β (Figure 5C). 24 h αS exposure also led to the appearance of apoptotic/picnotic nuclei (Figure 6A), which, contrary to what was observed in THP-1 monocytic cells, was consistent with a rough 20%-to-30% reduction of cell viability in 24 h αS-exposed compared with CTR THP-1-derived macrophages (Figure 6C). In line with this, a consistent reduction of the mRNA BCL-2/BAX ratio was also detected in 24 h αS-exposed compared with CTR THP-1-derived macrophages (Supplementary Figure S3).

### 3.6 αS uptake is associated with reduced autophagolysosomal markers in THP-1-derived macrophages

**FIGURE 7:**
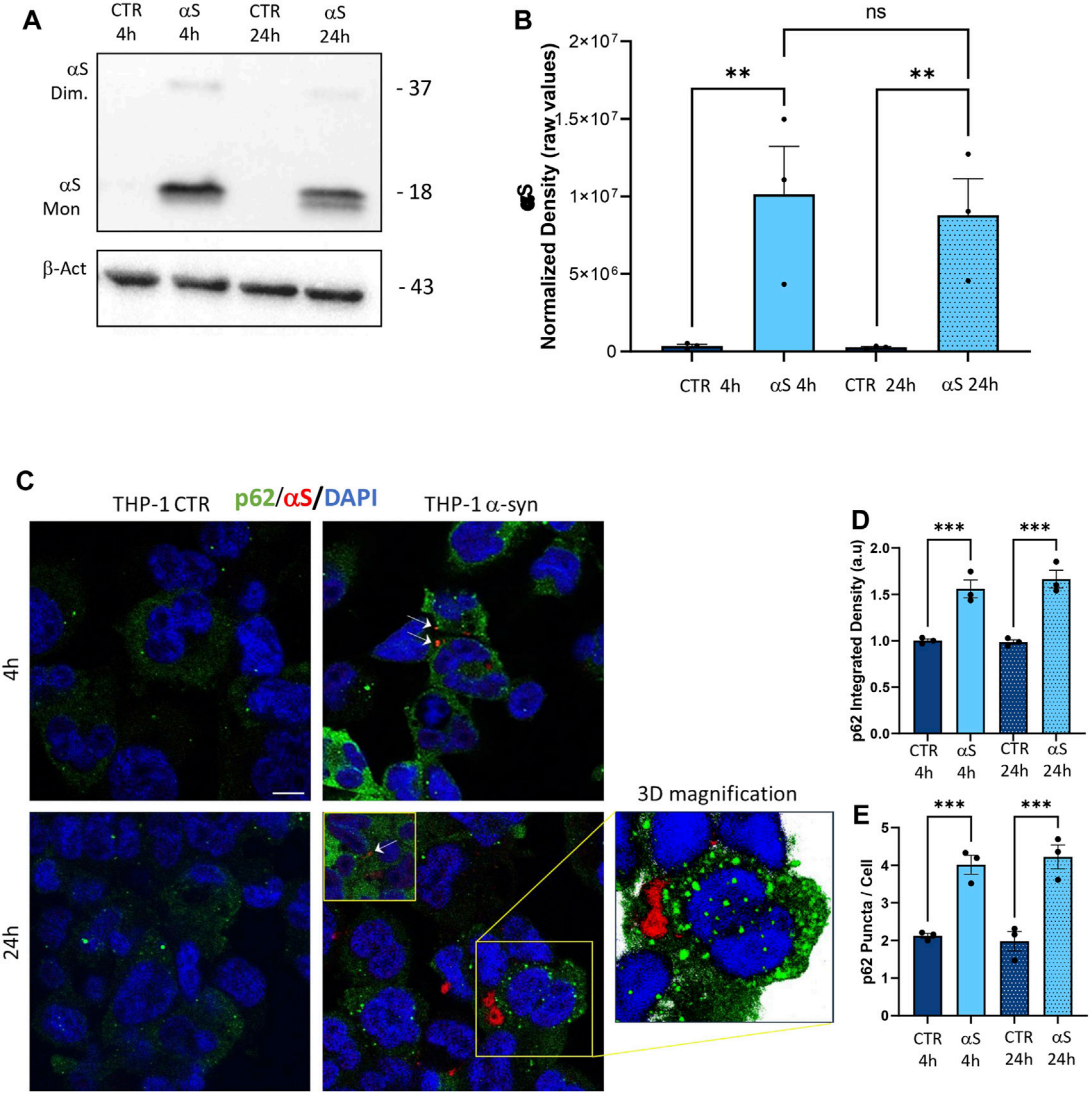
The uptake of αS is associated with p62 accumulation in THP-1-derived macrophages. **(A)** Representative Western blot for αS and β-actin in control (CTR) and αS-exposed THP-1-derived macrophages (MP, 4   h and 24 h). **(B)** Quantification of monomeric αS normalized to β-actin. Results are shown as normalized raw values corresponding to mean ± SEM. ***p < 0.001; ****p < 0.0001. **(C)** Representative Immunofluorescence against αS and p62 in control (CTR) and αS-exposed THP-1 monocytes for 4 h and 24 h. Arrows point at αS-p62 co-localizing puncta. Bars correspond to 10 μm. **(D, E)** Quantification of p62 integrated fluorescence density, and p62 puncta/cell in control (CTR) and αS-exposed THP-1-derived macrophages (MP) at 4 h and 24 h. Results are shown as mean ± SEM. ***p < 0.001; ****p < 0.0001.

To assess αS internalization and the autophagy/lysosomal dynamics in THP-derived macrophages, we performed a series of immunofluorescence, western blot, and gene expression analyses. Similarly to what was observed in THP-1 monocytic cells, THP-1-derived macrophages effectively internalized exogenous αS (Figure 7A–C), which accumulated predominantly as monomeric species (Figure 7A, B). Two interesting phenomena were observed in THP-1 derived macrophages contrary to THP-1 monocytic cells: i) endogenous αS protein was not detected in THP-1-derived macrophages (Figure 7A, C), suggesting that PMA-induced differentiation goes along with αS downregulation, as confirmed at mRNA level as well (Supplementary Figure S4); and ii) no statistically significant differences were detected in 4 h αS compared with 24 αS THP-1-derived macrophages in terms of intracellular αS amount, despite a tendency to decrease which is probably due to cell death (Figure 7B). This suggests the absence of an effective clearance mechanism to cope with intracellular αS overload in THP-1-derived macrophages, ultimately making them susceptible to αS-induced cell toxicity. Indeed, in THP-1-derived macrophages, exogenous αS staining showed a variously-sized, punctuate pattern reminiscent of protein inclusions that occasionally colocalized with the autophagy adaptor protein and substrate p62 (Figure 7C). Most importantly, αS exposure at 4 h and 24 h led to a significant increase in both p62 staining (Figure 7D), and p62 puncta/cell (Figure 7E), suggesting impaired turnover/clearance of p62.

**FIGURE 8:**
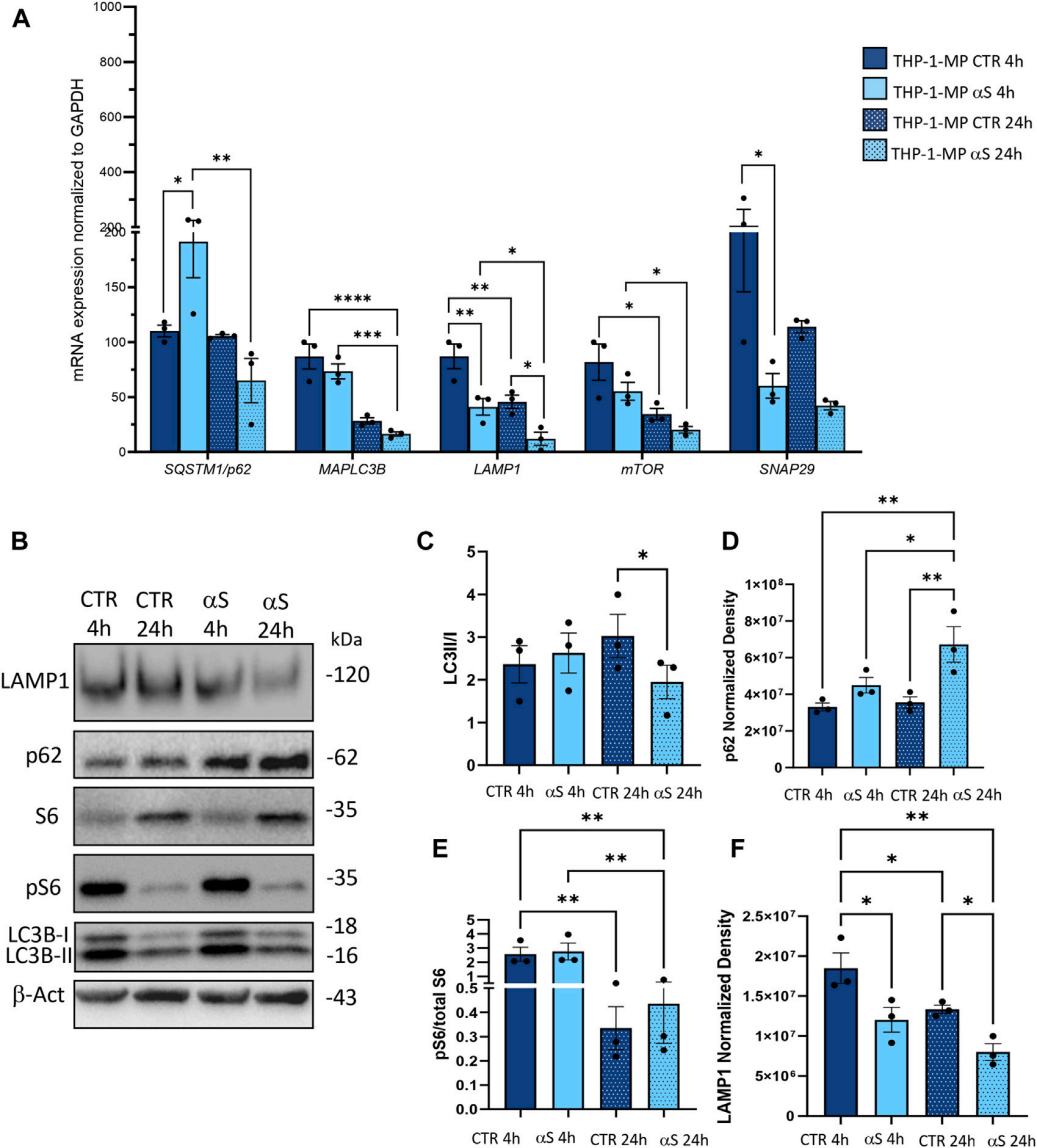
αS impairs autophagy-lysosomal markers in THP-1-derived macrophages. **(A)** Real time RT-qPCR analysis of autophagy-related genes in control (CTR) and αS-exposed THP-1-derived macrophages (4 h and 24 h). Values were normalized to *GAPDH* and are shown as mean ± SEM. *p < 0.05; **p < 0.01; ***p < 0.001; ****p < 0.0001. **(B)** Representative Western Blot against LC3II, LC3I, pS6, S6, p62, LAMP1 and β-actin in control (CTR) and αS-exposed THP-1-derived macrophages (MP, 4 h and 24 h). **(C–F)** Quantification of LC3II/I **(C)**, p62 **(D)**, LAMP1 **(E)**, and pS6/S6 **(F)**. Normalization was performed against β-actin, and results are shown as normalized raw values corresponding to mean ± SEM. *p < 0.05; **p < 0.01.

**FIGURE 9:**
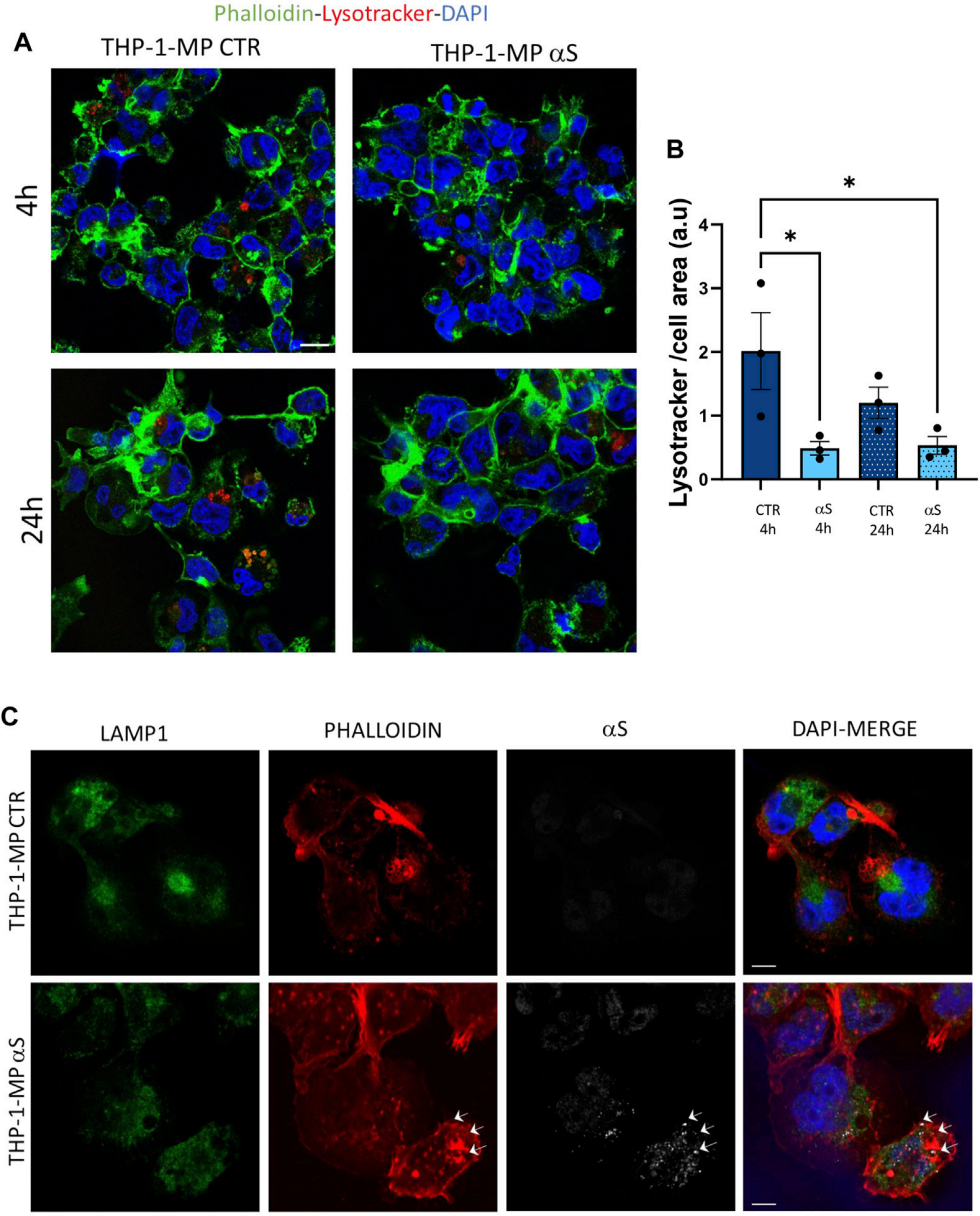
αS reduces lysosomal staining and co-localizes with F-actin in THP-1-derived macrophages. **(A)** Representative image of immunofluorescence staining with LysoTracker and F-actin (phalloidin) in control (CTR) and αS-exposed THP-1-derived macrophages (4 h and 24 h). **(B)** LysoTracker quantification in control (CTR) and αS-exposed THP-1-derived macrophages (4 h and 24 h). Values are shown as mean ± SEM. *p < 0.05. **(C)** Representative image of combined immunofluorescence for αS, LAMP1, and F-actin (phalloidin) in control (CTR) and αS-exposed THP-1-derived macrophages (4  h and 24 h). Arrows point at αS-phalloidin co-localization. Bars correspond to 10 μM.

To gain deeper insights into the effects of αS upon the autophagy-lysosomal pathway, we next examined mRNA and protein expressions of the same autophagy-related mediators that were measured in THP-1 monocytes. Similarly to what observed in THP-1 monocytes, 4 h αS exposure upregulated mRNA expression of the autophagy adaptor SQSTM1/p62 in THP-1-derived macrophages (Figure 8A); however, it significantly decreased mRNA expression of LAMP1, and SNAP29 compared with CTR macrophages (Figure 8A). Downregulation of LAMP1, and a downward trend for SNAP29 persisted at 24 h αS exposure compared with 24 h CTR (Figure 8A). Furthermore, mRNA expression of MAPLC3B, LAMP1, and mTOR was lower in 24 h αS- compared to 4 h αS-exposed THP-1-derived macrophages (Figure 8A). mRNA expression of LAMP1, and mTOR was also reduced in 24 h vs. 4 h CTR THP-1-derived macrophages, probably due to the progressive metabolic rewiring that takes places following PMA withdrawal in these cells. Western Blot analysis showed that, contrary to what observed in THP-1 monocytic cells, 24 h αS significantly decreased LC3II/I protein ratio (Figure 8C) meanwhile increasing p62 protein compared to CTR THP-1-derived macrophages (Figure 8D). LAMP1 protein time-dependently decreased in both experimental conditions while αS exposure at both 4 h and 24 h consistently reduced LAMP1 protein compared with CTRs (Figure 8E). Timing of PMA withdrawal produced a decrease in both mRNA expression of mTOR, and pS6/totalS6 protein ratio (24 h vs. 4 h), while αS exposure itself did not significantly alter pS6/totalS6 ratio at either time points, despite an upward trend being observed at 24 h compared with CTR THP-1 derived macrophages (Figure 8F). In line with the early reduction of LAMP1 by αS, LysoTracker staining was also decreased in 4 h αS-exposed compared to CTR THP-1-derived macrophages (Figure 9A, B). Again, exogenous internalized αS, while producing an evident qualitative decrease in LAMP1 staining, moderately co-localized with F-actin but not with LAMP1 in THP-1-derived macrophages (Figure 9C). These data suggest that, opposite to what observed in THP-1 monocytes, αS reduces autophagy/lysosomal markers in THP-1-derived macrophages via mTOR-independent mechanisms potentially including impaired LC3 lipidation/autophagosome maturation, autophagosome-lysosome fusion, impaired lysosomal efficacy, and aberrant interaction with cytoskeleton proteins, which eventually occludes the clearance of p62 and αS.

**FIGURE 10:**
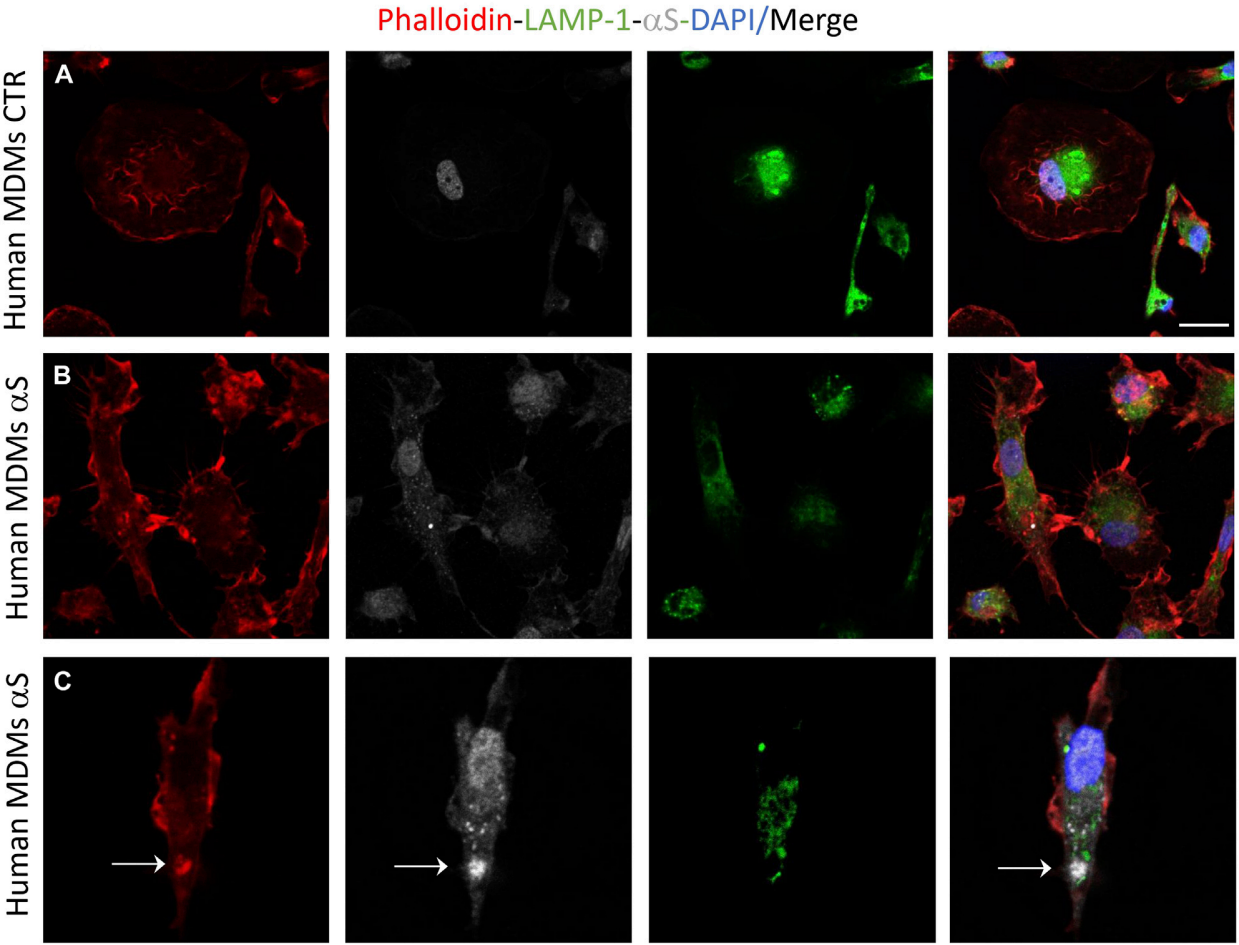
Human monocyte-derived macrophages (MDMs) take-up exogenous αS. Representative image of combined immunofluorescence for αS, LAMP1, and F-actin (phalloidin) in control (CTR, **A**) and αS-exposed, human MDMs (24 h, **B,C**).

Preliminary results on primary human monocyte-derived macrophages (MDMs) showed that αS is internalized by such cells where it appeared as cytosolic puncta Figure 10). Even if still low, constitutive expression of αS with a prominent nuclear localization was also detected within such cells (Figure 10A), contrary to THP-1-derived macrophages. In primary human MDMs, 24 h αS exposure produced a qualitative decrease in LAMP1 immunostaining meanwhile fostering cytoskeleton rearrangements resulting in a hyper-branched cell morphology, reminiscent of reactive macrophages. Again, in human MDMs, αS co-localized with F-actin clusters but not LAMP1 (Figure 10C).

### 3.7 αS-induced alterations of autophagy in THP-1-derived macrophages are associated with impaired lipid droplets and altered expression of cholesterol genes

**FIGURE 11:**
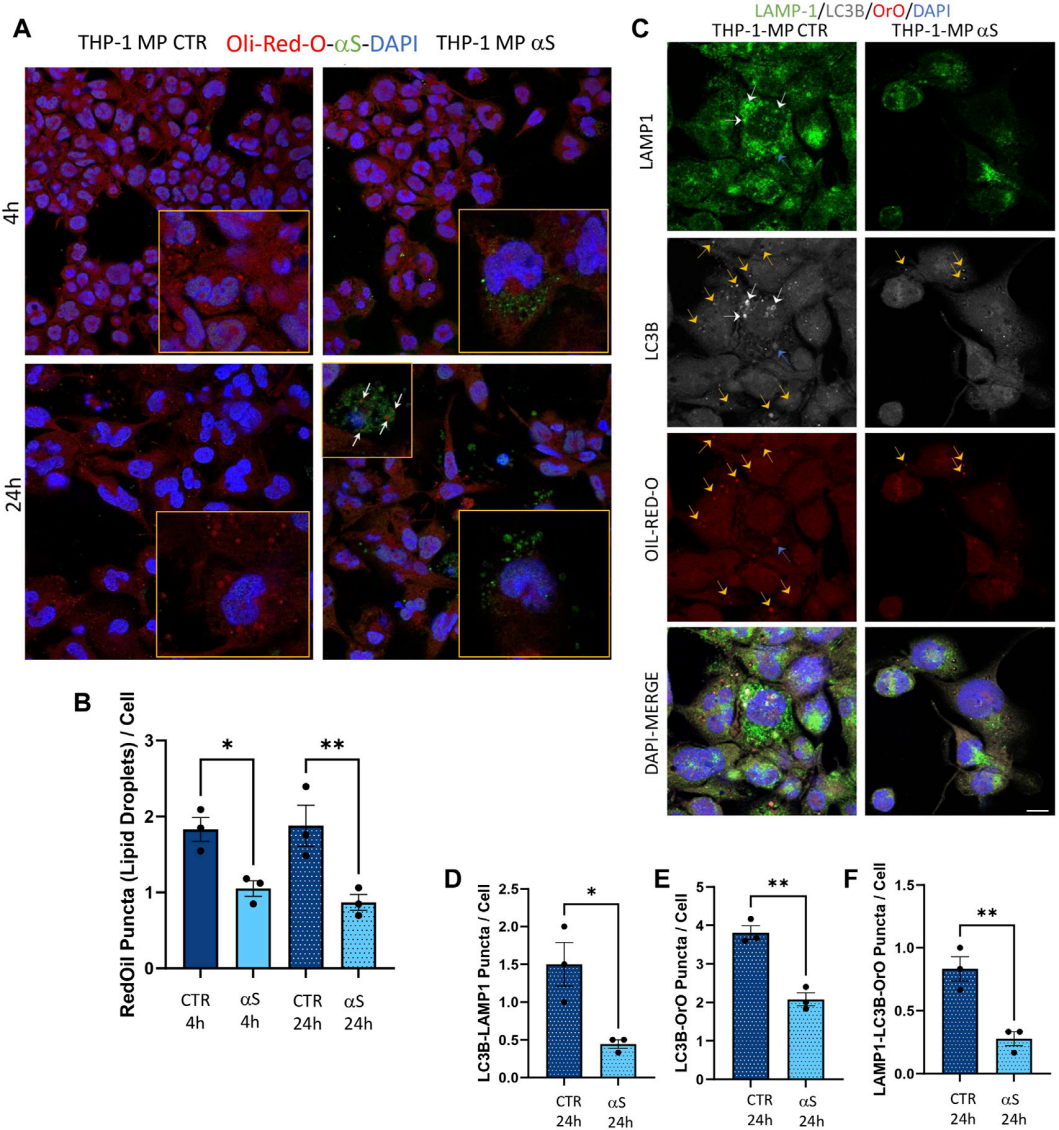
αS-induced autophagy perturbation in THP-1-derived macrophages is associated with altered lipid homeostasis. **(A)** Representative immunofluorescence of combined staining with Oil-Red-O (OrO) and αS in control (CTR) and αS-exposed THP-1-derived macrophages (4 h and 24 h). Arrows point at αS-OrO co-localization. Bars correspond to 10 μM. **(B)** Oil-Red-O puncta (lipid droplet) quantification in control (CTR) and αS-exposed THP-1-derived macrophages (4 h and 24 h). Values are shown as mean ± SEM. *p < 0.05, **p < 0.01. **(C)** Representative immunofluorescence of combined staining with Oil-Red-O, LC3B, LAMP1 in control (CTR) and αS-exposed THP-1-derived macrophages (MP, 24 h). White arrows point at LC3B-LAMP1 co-localization. Yellow arrows point at LC3B-Oil-red-O co-localization. Blue arrows point at LC3B-Oil-red-O-LAMP1 co-localization. Bars correspond to 10 μM. D-F. Quantification of LC3B-LAMP1 **(D)**, LC3B-Oil-red-O **(E)**, and LC3B-Oil-red-O-LAMP1 co-localizing puncta **(F)** in control (CTR) and αS-exposed THP-1-derived macrophages (MP, 24 h). Values are shown as mean ± SEM. *p < 0.05, **p < 0.01.

As alterations in intracellular lipid metabolism are associated with both macrophage inflammation and defects in degradative organelles, we next assessed if αS-induced reduction of autophagy-lysosomal markers associates with altered lipid dynamics in THP-1-derived macrophages. Results were unexpected, showing that αS consistently reduces lipid droplets’ (LDs) accumulation, as assessed through Oil-Red-O staining, in THP-1-derived macrophages (Figures 11A, B). LDs’ reduction was mostly evident in cells containing high levels of exogenous αS, including αS-filled cells undergoing cell death (Figure 11A). Combined immunofluorescence analysis for Oil-Red-stained LDs, LC3B, and LAMP1 (Figure 11C) showed that 24 h αS-exposure reduces the colocalization of LC3B and LAMP1 (Figure 11D), as well as the colocalization of LDs and LC3B (Figure 11E), and that of LDs, LCB, and LAMP1 (Figure 11F). These effects were associated with the modulation of cholesterol pathway genes’ expression, namely downregulation of the cholesterol efflux gene ABCG1 in 4 h αS-exposed cells, and upregulation of CAV-1, LXR, and XBP1 in 24 h αS-exposed compared with CTR THP-1-derived macrophages (Supplementary Figure S5). Instead, OSBP mRNA expression showed an upward, though not statistically significant trend following 4 h and 24 h αS exposure compared with respective CTR THP-1-derived macrophages (Supplementary Figure S5). Collectively, these data suggest that αS-induced alterations in lipid metabolism, including LDs formation and cholesterol pathway, might in turn contribute to impairing autophagy/lysosomal system via defective autophagosome, lysosome, and/or autolysosome formation/maturation.

### 3.8 αS impairs phagocytosis and the clearance of phagocytosed cargo in THP-1-derived macrophages

**FIGURE 12:**
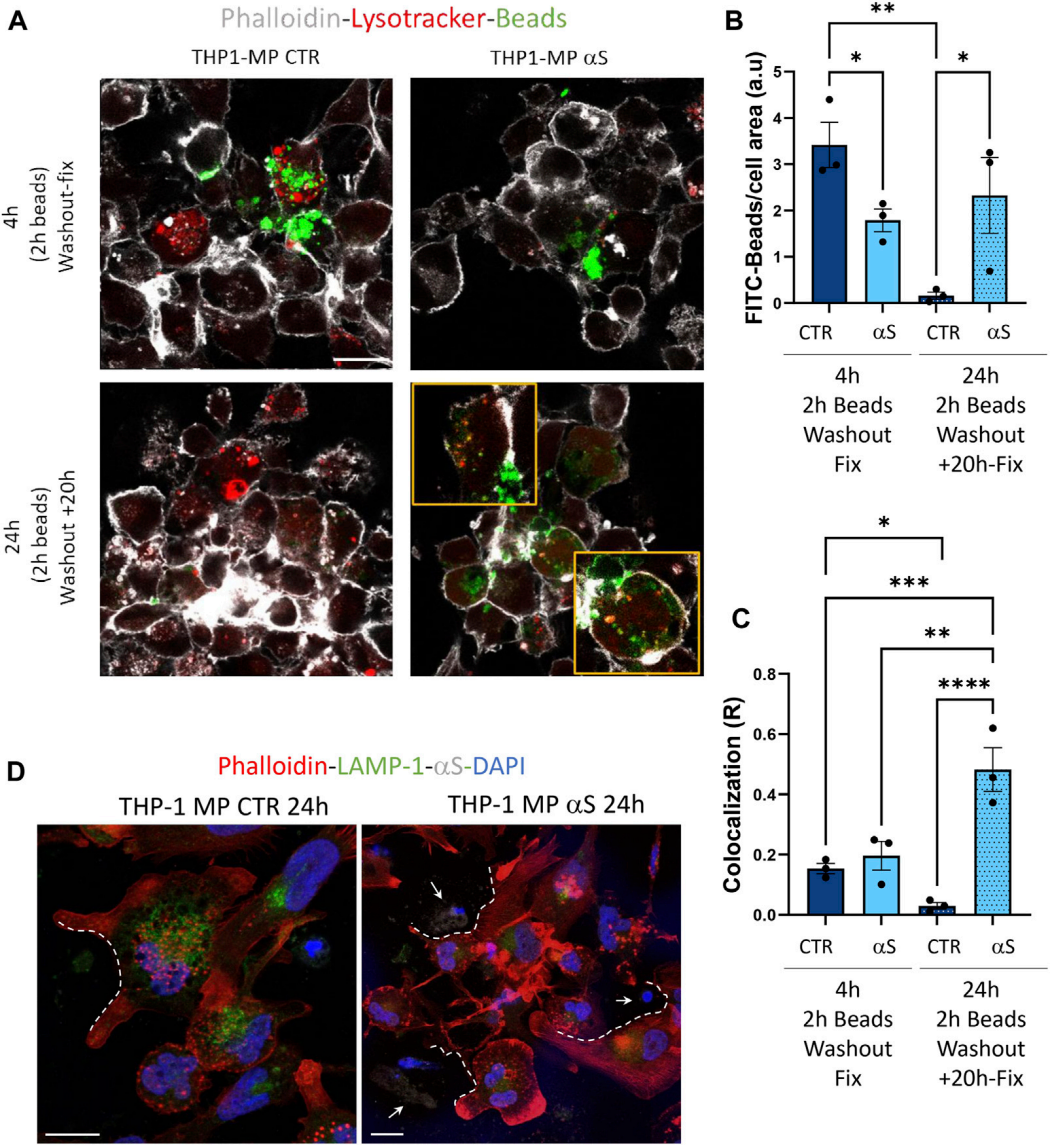
αS impairs phagocytosis and lysosomal processing of phagocytosed cargo in THP-1-derived macrophages. **(A)** Representative immunofluorescence of control (CTR) and αS-exposed THP-1-derived macrophages (4 h) immediately after 2 h incubation with FITC-latex beads (4 h, Washout fix) and following an additional 20 h culturing after beads washout (24 h, Washout + 20 h). Cells were stained with LysoTracker and Phalloidin. Bars correspond to 10 μM. **(B, C)**. Quantification of FITC-beads **(B)** and LysoTracker-FITC R colocalization **(C)** in control (CTR) and αS-exposed THP-1-derived macrophages in the two experimental conditions (4 h, Washout fix, and 24 h, Washout + 20 h). Values are shown as mean ± SEM. *p < 0.05, **p < 0.01, ***p < 0.001. ****p < 0.0001. **(D)** Combined immunofluorescence for αS, LAMP1, and F-actin (phalloidin) in control (CTR) and αS-exposed THP1-derived macrophages (MP, 24 h). White dashed lines indicate phagocytic-like protrusions, and arrows point at αS-filled dead cells. Bars correspond to 10 μM.

Finally, we assessed whether αS functionally alters THP-derived macrophages by measuring their phagocytic capacity and the intracellular fate of phagocytosed cargo. In line with previous evidence in both microglia and primary human macrophages (Gardai et al., 2013; Haenseler et al., 2017), our results showed that αS reduces the phagocytic capacity of THP-1-derived macrophages (Figure 12A, B). In fact, following 2 h incubation with FITC-IgG latex beads, 4 h αS-exposed THP-1-derived macrophages showed a two-fold reduction of FITC signal corresponding to phagocytosed beads (Figure 12A, B). At such a time-point, only occasional colocalization of beads and LysoTracker-marked lysosomes was detected in both αS-exposed and CTR cells, without significant differences (Figure 12A, C). Remarkably, following beads washout and culturing of cells for additional 20 h, FITC signal was almost completely absent in CTR-THP-1-derived macrophages, suggesting effective processing/dimming of FITC dye in acidic organelles (Figure 12A–C). Instead, the same amount of initially phagocytosed beads (FITC signal) was still present in αS-exposed THP-1-derived macrophages even after additional 20 h culturing following beads washout (Figure 12A, B). Such FITC-stained phagocytosed cargo markedly filled the LysoTracker-stained organelles, resulting in a co-localization pattern reminiscent of engulfed/stagnant lysosomes (Figure 12A, C). These data suggest that αS impairs phagocytosis and lysosomal processing of phagocytosed cargo. Combined staining for F-actin, αS, and LAMP1 showed the occasional presence of extracellular material which clustered near/around the phagocytic-like protrusions of CTR THP-1-derived macrophages (Figure 12D). In 24 h αS-exposed THP-1 derived macrophages, several αS-filled dying/dead cells were instead observed, which clustered near aberrantly appearing phagocytic-like protrusions of THP-1-derived macrophages, reminiscent of impaired efferocytosis (Figure 12D).

## 4 Discussion

In the present study we add to previous evidence that extracellular αS is effectively internalized by THP-1 monocytes, and THP-1-derived macrophages (Figures 1, 7) (Haenseler et al., 2017; Liu et al., 2022; Moriya et al., 2022), to show how this produces consistent, though different biological and functional effects in the two cell types. Indeed, different time-related intracellular αS dynamics were observed between the two cell types, which were in turn associated with diverse effects upon cell viability, inflammatory profile, and markers of the autophagy-lysosomal pathway. Firstly, in THP-1 monocytic cells, a decrease in intracellular αS amount was detected at 24 h compared with 4 h exposure. Coupled with the lack of toxic effects induced by excess αS (1 μM) at 4 h or 24 h, these data suggest that THP-1 cells well-tolerate αS overload, probably by diluting and/or degrading the cargo of internalized protein (Figure 1A–D). Such an effect was not observed in THP-1-derived macrophages (Figure 7B), which were instead intoxicated by prolonged αS exposure (24 h, Figure 6, Supplementary Figure S3). These data are in line with the results of a recent study (Haenseler et al., 2017). Again, in both cell-types, excess αS induced an apparently similar pro-inflammatory profile by potentiating the gene transcription and secretion of various cytokines and chemokines. In detail, in THP-1 monocytes, upregulation of most secreted cytokines/chemokines occurred already after short αS exposure (4 h) (Figure 2). Indeed, the conditioned medium from αS-THP-1 monocytes potently attracted unstimulated cells (Figure 3). Confirming previous evidence, our data suggest that intracellular αS accumulation induces a pro-inflammatory extracellular milieu which serves as a chemoattractant for circulating monocytes (Klegeris et al., 2008; Allen Reish and Standaert, 2015; White et al., 2018; Grozdanov et al., 2019; Liu et al., 2022; Pellegrini et al., 2022; Su et al., 2022). Remarkably, such a pro-inflammatory scenario was not reproduced for all cytokines/chemokines at 24 h of αS exposure (Figure 2C). In detail, the concentrations of MCP-1/CCL2, MIP-1b/CCL4, and TNF-α were lower in 24 h-compared with 4 h-exposed αS. Similar different effects of 4 h vs. 24 h αS exposure were observed for the extra- and intracellular dynamics of the CCL2 receptor (CCR2) in THP-1 monocytes (Supplementary Figure S2). In fact, 4 h αS exposure readily decreased cell surface CCR2 while increasing intracellular/total CCR2 ratio compared with CTR, suggesting rapid monocytic activation and receptor internalization which follows up ligand binding. However, no further differences were observed at prolonged αS exposure (24 h) vs. either 24 h CTR or 24 αS-exposed cells, suggesting that CCR2 recycling back to the plasma membrane takes place at such a time-point (Supplementary Figure S2). As CCR2 dynamics involve a delicate balance between agonist-dependent CCR2 sensitivity, and constitutive chemokine scavenging activity of the receptor (Volpe et al., 2012; Shroka et al., 2023), it is likely that prolonged αS exposure triggers a compensatory cellular mechanism which contributes to reducing CCR2 responsiveness and/or CCL2 levels. Unveiling the molecular bases of such a compensatory mechanism, as documented, for instance, for TLR signalling via a feedback mechanism operated by SOCS3 and SHP phosphatases (Kubo et al., 2003), goes beyond the aim of the present study; however it deserves to be addressed by future studies. In our hands, CCR2 dynamics appeared unrelated to CCL2-dependent chemotaxis, as αS-exposed THP-1 cells displayed unaltered, and reduced chemotaxis at 4 h and 24 h, respectively (Figure 3). Interestingly, αS-exposed cells also showed a time-dependent alteration in their spontaneous (FBS-driven) migration capacity (Figure 3), suggesting that intracellular αS levels influence the mobility of monocytic cells in response to growth factors beyond cytokines/chemokines, which deserves further investigation as well.

Contrary to what was observed for the monocytic THP-1 cells, αS exposure in THP-1-derived macrophages produced a time-related intracellular accumulation (4 h), and subsequent (24 h) secretion of inflammatory cytokines/chemokines (IL-1β, IL-4, IL-6, IL-10, IL-17, G-CSF, GM-CSF, IFN-γ, MIP-1b/CCL4, and TNF-α) (Figures 5, 6). Such an effect included a marked upregulation of cytokines which were not altered by αS in the THP-1 monocytic counterpart, namely G-CSF, GM-CSF, and IFN-γ. Remarkably, our results suggest that autophagy might represent a major cellular mechanism underpinning the different effects of αS within monocytes and macrophages. In fact, the effects of αS were associated with induction of autophagy-lysosomal markers in THP-1 monocytic cells (Figure 4) and their suppression in THP-1-derived macrophages (Figures 7–10). In detail, the stimulating effects of αS upon the autophagy/lysosomal markers were mostly evident at 24 h exposure, when increased LC3II/I ratio was parallelled by a decrease in p62 protein, and the mTOR activity index pS6/total S6 protein ratio, along with an increase in LAMP1 at both mRNA and protein levels (Figure 4). These findings suggest that THP-1 monocytes respond to excess αS by reducing mTOR activity, to promote upregulation of autophagolysosome markers, and degradation of autophagy substrates. This might explain why reduced levels of internalized αS were detected at 24 vs. 4 h exposure, and why THP-1 monocytes well tolerate αS overload. Although the cytoplasmic cleanup function of autophagy is generally anti-inflammatory in most cell types, autophagy upregulation has been implicated in monocytes’ activation during the early stages of pathological events, and preventing the induction of autophagy in monocytes hinders their survival, differentiation and cytokine production (Zhang et al., 2012; Germic et al., 2019; Chen et al., 2023). Thus, autophagy activation following αS internalization in THP-1 monocytes may occur as a pro-survival/tolerance mechanism coping with stress-related inflammation, which in turn, is functionally associated with blunted CCL2 secretion and reduced chemotaxis. However, further studies employing pharmacological modulators or genetic manipulation of autophagy are needed to confirm such results, and to explore whether/how autophagy interferes with CCL2 release and CCR2 dynamics.

Notably, in THP-1-derived macrophages, 24 h αS significantly decreased LC3II/I ratio while increasing p62 protein, which went along with decreased LAMP1 levels detected already at 4 h of αS exposure both at protein and mRNA level (Figure 8). Supporting αS-induced lysosomal dysfunction, LysoTracker staining was also consistently decreased (Figure 9). However, the index of mTOR activity, namely the pS6/total S6 ratio was not significantly affected by αS exposure (Figure 8), suggesting that αS downregulates autophagy-lysosomal markers through an mTOR-independent mechanism. These results suggest that αS-loaded macrophages fail to overcome the detrimental effects of αS, with autophagy impairment potentially bridging reduced cell survival, and excess activation of pro-inflammatory pathways. Our data are in line with previous evidence that αS induces impairment of autophagy pathway and/or lysosome rupture in a variety of cell types, including microglia and macrophages (Freeman et al., 2013; Tu et al., 2021). Herein, we also detected significant αS-induced modulations of SQSTM1/p62, and LAMP1 mRNA expressions in both cell types, which suggests also a transcriptional, probably epigenetic effect on such targets. Importantly, in THP-1-derived macrophages, αS also produced an early downregulation of the autophagosome-lysosome fusion marker SNAP29 (Figure 8A), in line with both previous studies (Tang et al., 2021), and our data showing reduced co-localization of LC3 and LAMP-1 (Figure 11C, D). Again, in both THP-1-derived macrophages, and MDMs, we constantly detected a co-localization of αS and F-actin (Figures 9, 10), which deserves further investigations on the possible role of αS on cytoskeleton dynamics.

In our hands, the effects of αS in THP-1-derived macrophages were associated with reduced intracellular accumulation of LDs (Figure 11). This finding was unexpected at first glance, as excess LDs accumulation has been previously associated with both macrophage hyper-inflammation, and autophagy impairment (Rosas-Ballina et al., 2020). Reduced LDs might be due to increased lipophagy, impaired LD biogenesis, or excess lipid/cholesterol efflux (Robichaud et al., 2021). However, autophagy/lysosome-dependent lipolysis is unlikely to be our case, as αS itself reduced autophagolysosomal markers (LC3B and LAMP1, LysoTracker), as well as their co-localization with LDs (Figure 11). Again, mRNA expression of the cholesterol pathway genes CAV-1, CH25H, and LXR showed a downwards trend in 4 h αS-exposed vs. CTR THP-1-macrophages, with the cholesterol efflux/transporter gene ABCG1A being significantly decreased; contrariwise, most genes except for ABCG1A (namely, CAV-1, LXR, and XBP1) were significantly upregulated by 24 h αS exposure (Supplementary Figure S5). A consistent reduction of LDs was observed already at 4 h αS exposure and persisted at 24 h, which suggests that upregulation of specific cholesterol pathway genes detected at 24 h αS might represent a compensatory response to protein reductions. This mechanism seems inversely specular to what observed herein for pro-inflammatory genes and related encoded proteins. A plausible explanation to LDs impairment by αS stems from evidence showing that αS interacts with lipids on biological membranes to promote lipids extraction, or even lowering of cholesterol, an essential constituent of LDs (Emanuele et al., 2016; Alza et al., 2019; Lazarevic et al., 2022). Studies on the biological role of LDs showed that these organelles act as sinks that sequester various oxidized/toxic lipids and proteins to limit their availability for participation in signaling pathways that may cause cell damage and inflammation (Jarc and Petan, 2020). Again, a recent stream of evidence converges in that LDs, through their flow from-and-to the endoplasmic reticulum, are an essential source for autophagosome biogenesis and normal progression of autophagy (Shpilka and Elazar, 2015). Thus, while altering the integrity of biological membranes, αS might also impinge on the biogenesis of LDs, which might in turn fuel cell toxicity, inflammation, and impaired autophagy in macrophages. A recent study (Haenseler et al., 2017) showed that αS depletes intracellular cholesterol by promoting excess cholesterol efflux in iPSC-derived macrophages. This was in turn associated with impaired phagocytic capacity, which is in line with our results (Figure 12) and those documented in peritoneal macrophages (Gardai et al., 2013). Though in our hands αS early decreased the mRNA expression of the cholesterol transporter ABCG1A, we did not directly verify if impaired LDs are due to altered cholesterol efflux. However, herein we document that, besides impairing phagocytosis in THP-1-derived macrophages, αS compromises lysosomal processing of phagocytosed cargo probably by altering their acidification (Figure 12). In summary, our data suggest that αS bridges impaired lipid metabolism, defective autophagy/lysosomal system, and excess inflammation to alter macrophage biology by producing a status of macrophage exhaustion reminiscent of hypophagia.

Finally, it is worth of mentioning that, although low, endogenous αS was detectable in THP-1 monocytes (Figure 1) and it was abolished in THP-1-derived macrophages (Figure 7A, C; Supplementary Figure S4) but not in M-CSF-differentiated MDMs (Figure 10), suggesting that αS downregulation occurs downstream of PMA treatment. This is probably related to the different biological roles of αS and PMA on shared molecular targets, such as protein kinase C (PKC), and PKC-dependent regulation of vesicle trafficking, as documented in various biological contexts (Kim et al., 2010; Huang et al., 2018).

In summary, our findings suggest that monocytes and macrophages respond differently to intracellular αS accumulation in terms of cell survival, metabolism, and functions, which deserves to be further investigated for its implications in the pathobiology of inflammation and synucleinopathies such as PD. However, we wish to point out that our study is not devoid of limitations. First, undifferentiated THP-1 monocytes are immortalized, actively dividing cells, and as such, they own a different inherent metabolic profile compared with their differentiated counterpart. Second, our findings are mostly descriptive and observational, as we did not employ specific pharmacologic/genetic modulators of autophagy/lysosomal pathway to confirm that the effects of αS are indeed specifically related to stimulation or suppression of autophagy. This is important since autophagy substrates, including p62 and αS itself, are also degraded by the proteasome (Limanaqi et al., 2020; 2021a). Further studies on primary cells, and the use of combined administration with various autophagy modulators will be seminal to further clarify the biological role of αS in monocytes and macrophages in health and disease, which is currently under investigation in our lab.

## Data Availability

The original contributions presented in the study are included in the article/Supplementary Material, further inquiries can be directed to the corresponding authors.
