# Supplementary figures and images for: Alpha-synuclein shapes monocyte and macrophage cell biology and functions by bridging alterations of autophagy and inflammatory pathways
Source: Front Cell Dev Biol. 2024 Jul 5;12:1421360. doi: 10.3389/fcell.2024.1421360 (PMC11257978; doi:10.3389/fcell.2024.1421360)

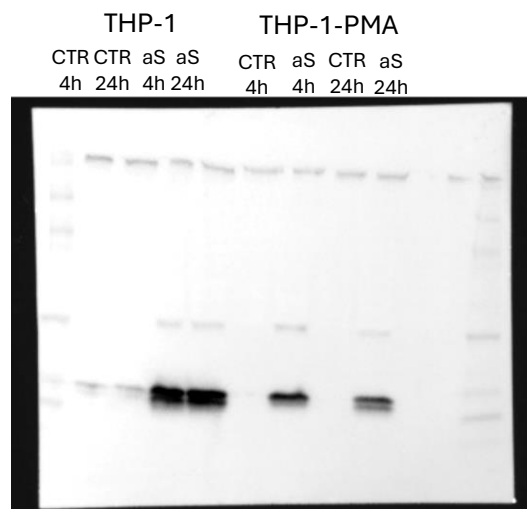

A-SYN

(Figure 1B, Figure 7A)

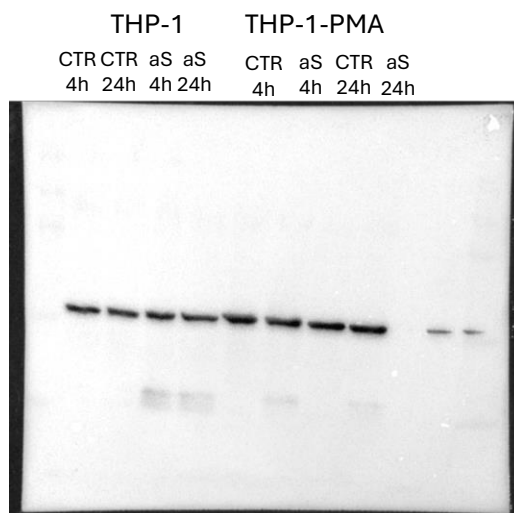

B-ACT

(Figure 1B, Figure 7A)

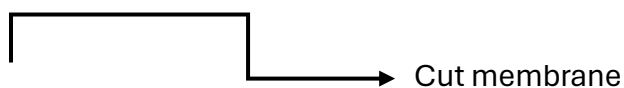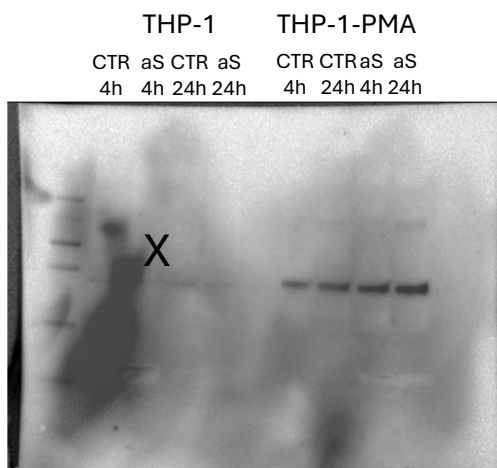

p62

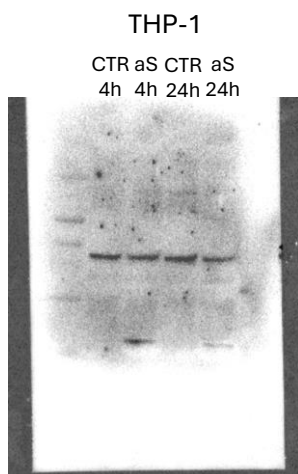

p62 (Figure 4B)

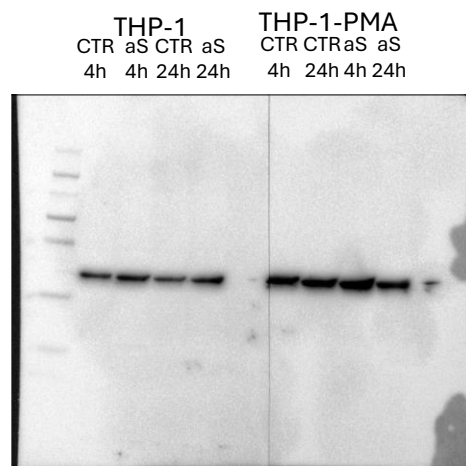

B-ACT

(Figure 4B)

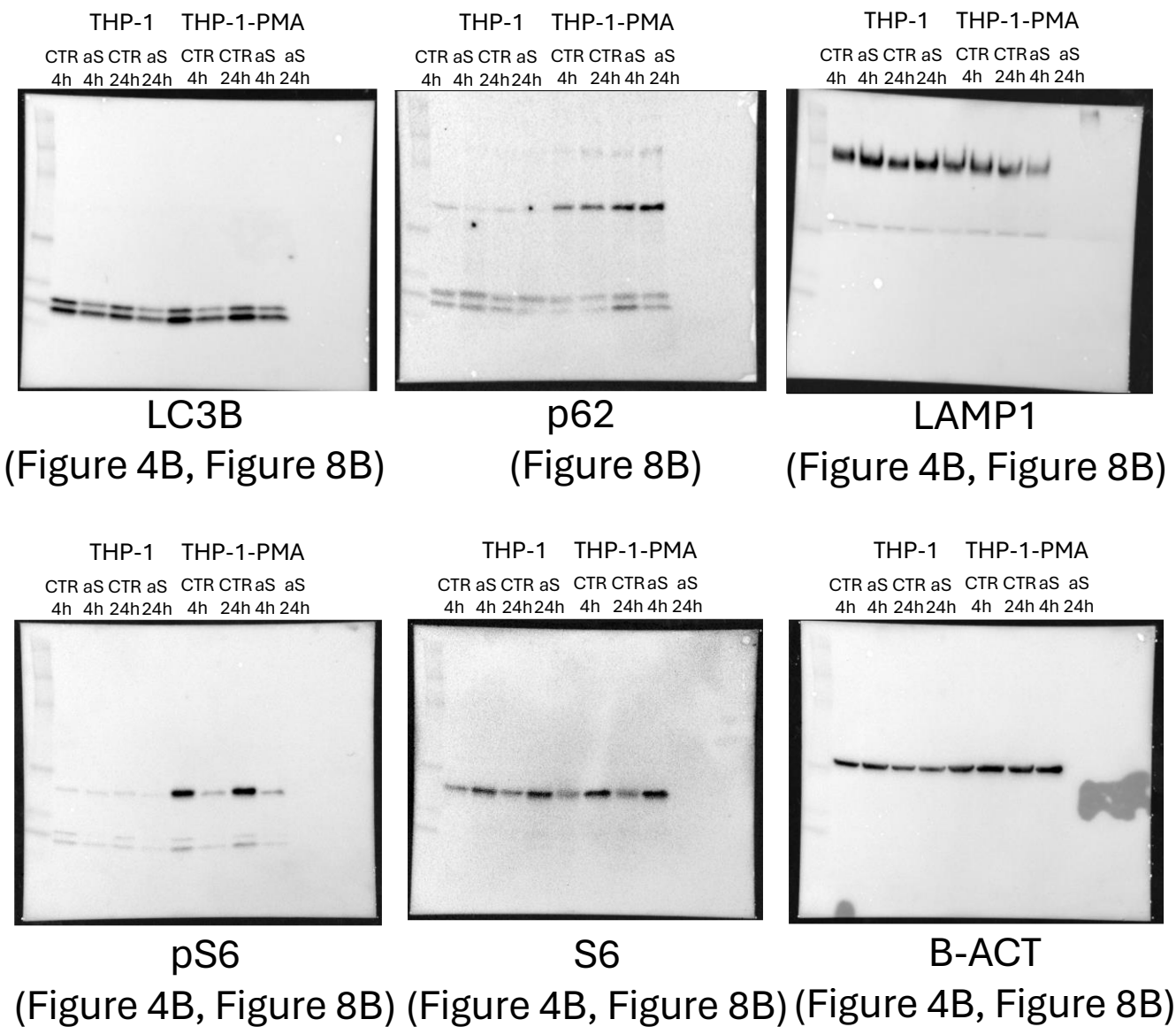

Supplement: Supplementary file 1 [file DataSheet2.PDF]
