# Supplementary material for: Alpha-synuclein shapes monocyte and macrophage cell biology and functions by bridging alterations of autophagy and inflammatory pathways
Source: Front Cell Dev Biol. 2024 Jul 5;12:1421360. doi: 10.3389/fcell.2024.1421360 (PMC11257978; doi:10.3389/fcell.2024.1421360)
Supplement: Supplementary file 2 [file DataSheet1.pdf]

Supplementary Material

Alpha-synuclein shapes monocyte and macrophage cell biology and functions by bridging alterations of autophagy and inflammatory pathways.

Fiona Limanaqi[1†]*, Silvia Zecchini[1†], Pasquale Ogno[1], Valentina Artusa[2], Claudio Fenizia[2], Irma Saulle[2], Claudia Vanetti[1], Micaela Garziano[2], Sergio Strizzi[1], Daria Trabattoni[1], Mario Clerici[2,3], Mara Biasin[1]*

[1]Department of Biomedical and Clinical Sciences, University of Milan, Via G.B. Grassi, Milan, Italy
[2]Department of Pathophysiology and Transplantation, University of Milan, Via Francesco Sforza, Milan, Italy
[3]IRCCS Fondazione Don Carlo Gnocchi, 20148 Milan, Italy
[†]These authors equally contributed.
***Correspondence**:

Mara Biasin mara.biasin@unimi.it; Fiona Limanaqi fiona.limanaqi@unimi.it

Supplementary Figures

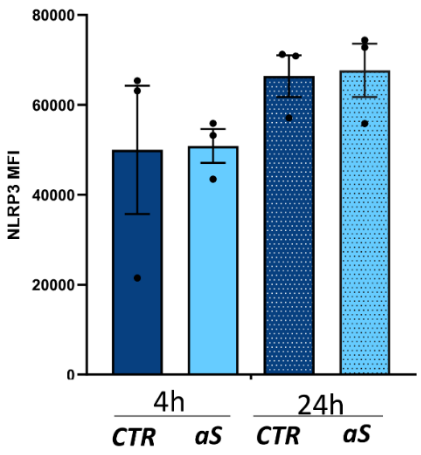

Supplementary Figure S1. αS does not alter intracellular NLRP3 levels in THP-1 cells. **A.** Flow cytometry-based quantification of NLRP3 protein in CTR and αS-exposed THP-1 cells (4h and 24h). Results are shown as mean fluorescence intensity (MFI)±SEM.

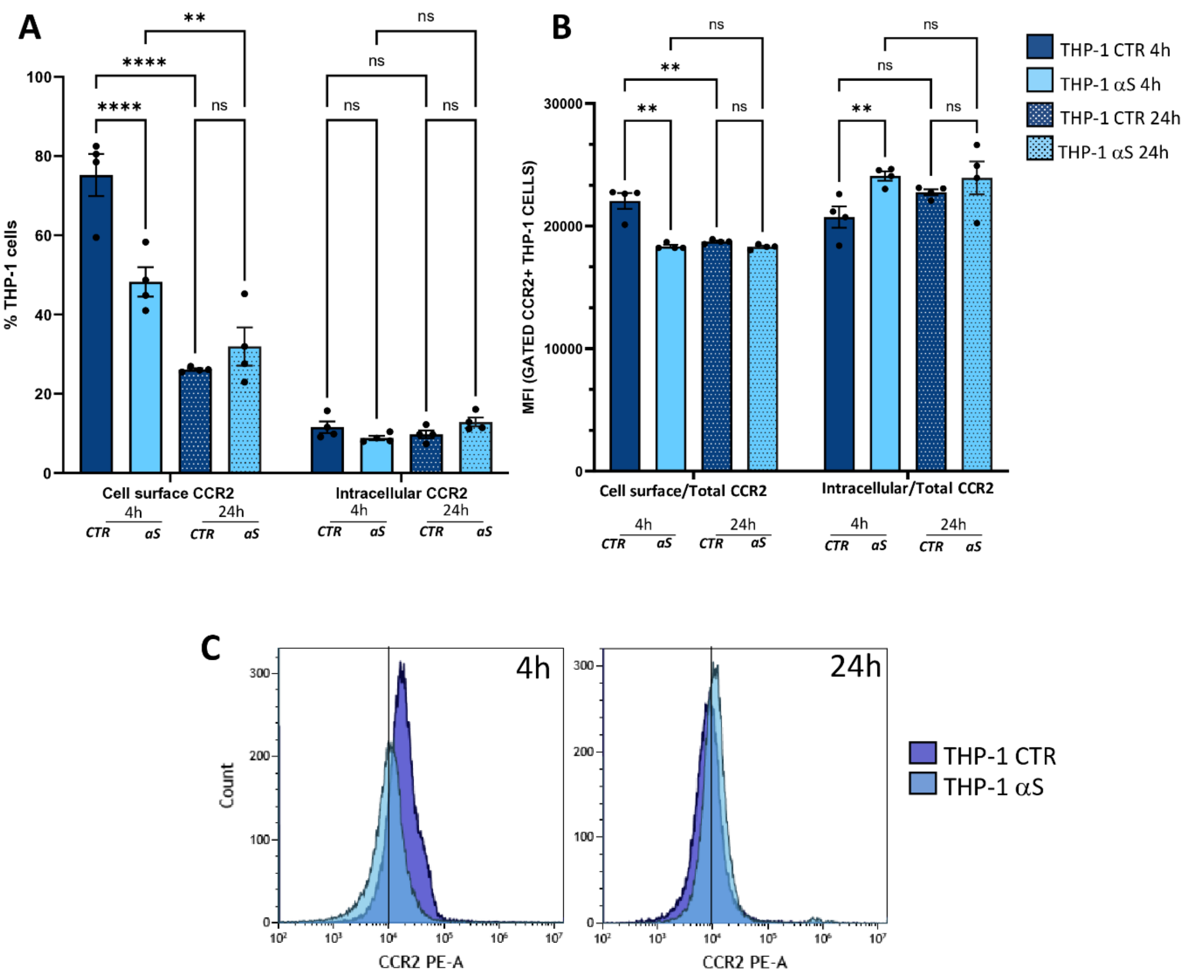

Supplementary Figure S2. 4h αS exposure alters extracellular and intracellular CCR2 dynamics in THP-1 cells. **A.** Flow cytometry-based quantification of THP-1 cells stained for cell surface or intracellular CCR2 in CTR and αS-exposed THP-1 cells (4h and 24h). Results are shown as the mean percentage of THP-1 cells ±SEM. **p<0.01; **** p<0.0001. **B**. Ratio of mean fluorescence intensity (MFI) of cell surface/total CCR2, and intracellular/total CCR2 in CTR and αS-exposed THP-1 cells (4h and 24h). Gating was performed on CCR2-positive THP-1 cells. Results are shown as mean±SEM. **p<0.01. **C.** Representative images of cell surface CCR2 MFI in CTR and αS-exposed THP-1 cells (4h and 24h).

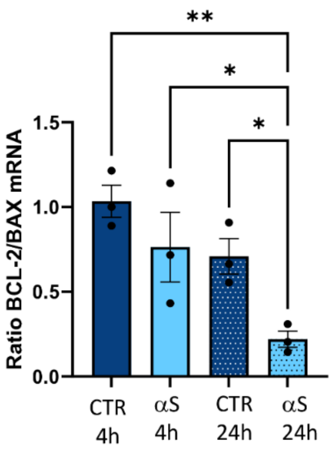

Supplementary Figure S3. 24h αS exposure decreases BCL-2/BAX mRNA ratio in THP-1-derived macrophages. **A.** Ratio of BCL-2/BAX mRNAs quantified through real time RT-qPCR analysis and normalized to *GAPDH* in CTR and αS-exposed THP-1-derived macrophages (4h and 24h). Values represent mean±SEM. *p<0.05; **p<0.01.

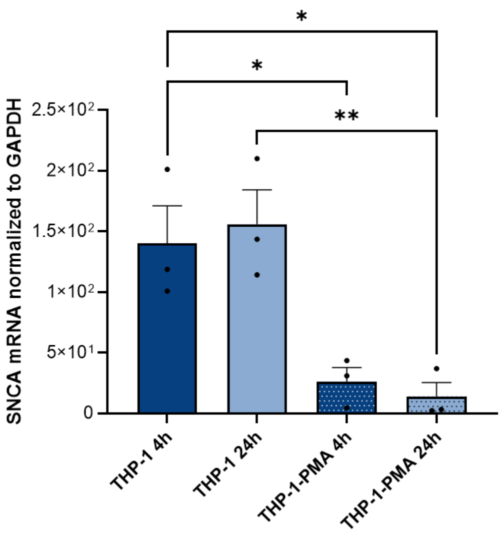

Supplementary Figure S4. PMA-induced differentiation of THP-1 monocytes-to-macrophages goes along with downregulation of αS mRNA expression. *Synuclein-alpha (SNCA)* mRNA expression was quantified through Real time RT-qPCR and normalized to *GAPDH* in THP-1 monocytic cells, and THP-1-derived macrophages (4h and 24h). Cq values in THP-1 monocytic cells were low, though above the threshold (range between 30 and 34) while in THP-1-derived macrophages Cq values were below threshold (from >35 to non-detectable, which were randomly assigned a value of 40). Relative values represent mean±SEM. *p<0.05; **p<0.01.

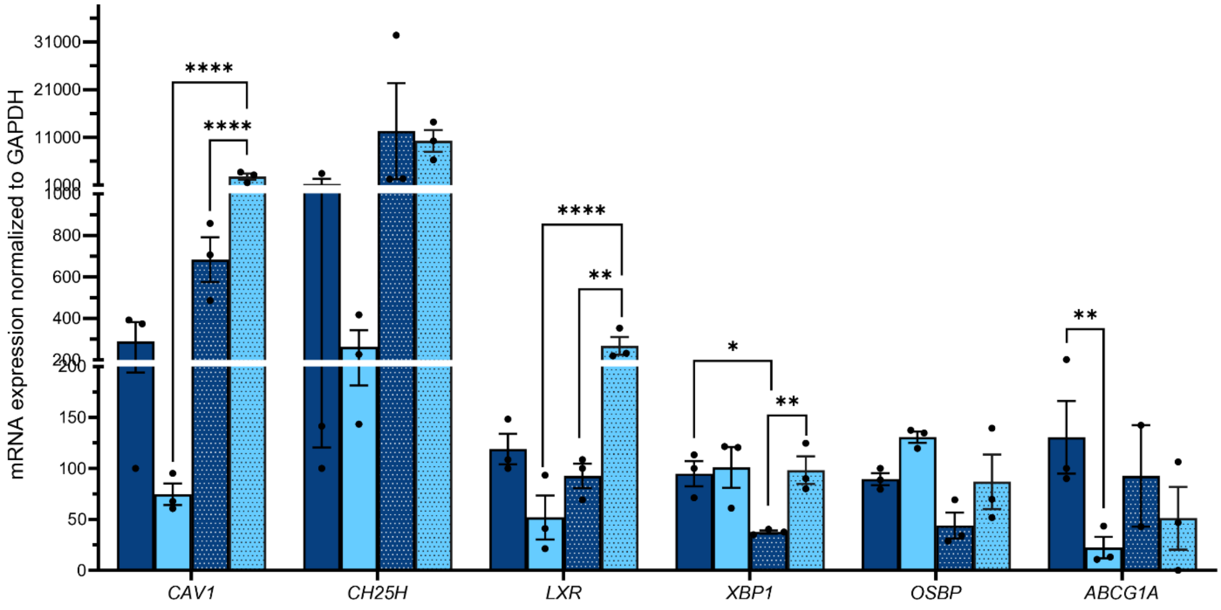

Supplementary Figure S5. αS alters mRNA expression of cholesterol pathway genes in THP-1-derived macrophages. Real time RT-qPCR analysis of cholesterol pathway genes in control (CTR) and αS-exposed THP-1-derived macrophages (4h, solid fill, and 24h, patterned fill). Values were normalized to *GAPDH* and are shown as mean±SEM. *p<0.05, **p<0.01, ****p<0.0001.
